# The Pathway to Detangle a Scrambled Gene

**DOI:** 10.1371/journal.pone.0002330

**Published:** 2008-06-04

**Authors:** Matthias Möllenbeck, Yi Zhou, Andre R. O. Cavalcanti, Franziska Jönsson, Brian P. Higgins, Wei-Jen Chang, Stefan Juranek, Thomas G. Doak, Grzegorz Rozenberg, Hans J. Lipps, Laura F. Landweber

**Affiliations:** 1 Institute of Cell Biology, University Witten/Herdecke, Witten, Germany; 2 Ecology & Evolutionary Biology, Princeton University, Princeton, New Jersey, United States of America; 3 Institute of Advanced Computer Science, Leiden University, Leiden, The Netherlands; Centre for DNA Fingerprinting and Diagnostics, India

## Abstract

**Background:**

Programmed DNA elimination and reorganization frequently occur during cellular differentiation. Development of the somatic macronucleus in some ciliates presents an extreme case, involving excision of internal eliminated sequences (**IESs**) that interrupt coding DNA segments (macronuclear destined sequences, **MDSs**), as well as removal of transposon-like elements and extensive genome fragmentation, leading to 98% genome reduction in *Stylonychia lemnae*. Approximately 20–30% of the genes are estimated to be scrambled in the germline micronucleus, with coding segment order permuted and present in either orientation on micronuclear chromosomes. Massive genome rearrangements are therefore critical for development.

**Methodology/Principal Findings:**

To understand the process of DNA deletion and reorganization during macronuclear development, we examined the population of DNA molecules during assembly of different scrambled genes in two related organisms in a developmental time-course by PCR. The data suggest that removal of conventional IESs usually occurs first, accompanied by a surprising level of error at this step. The complex events of inversion and translocation seem to occur after repair and excision of all conventional IESs and via multiple pathways.

**Conclusions/Significance:**

This study reveals a temporal order of DNA rearrangements during the processing of a scrambled gene, with simpler events usually preceding more complex ones. The surprising observation of a hidden layer of errors, absent from the mature macronucleus but present during development, also underscores the need for repair or screening of incorrectly-assembled DNA molecules.

## Introduction

DNA elimination and reorganization occur in a variety of differentiating eukaryotic cells. Genome reduction may involve either whole chromosomes, as in differentiating cells of *Sciara coprophila* [1] or parts of chromosomes, as in nematodes or *Cyclops* [2]–[4]. The best studied example of specific DNA excision and rearrangement is the processing of immunoglobulin and T-cell receptor genes in mammalian cells [5], [6]. The mechanisms of all these rearrangements are not completely understood, but most cases probably involve recombination [7], [8], similar to mating type switching in yeast [9], [10] or antigenic variation in trypanosomes [11], [12]. The most elaborate known form of DNA rearrangement occurs during macronuclear development in ciliated protists, which provide model systems to study programmed DNA elimination, fragmentation, and reorganization during development [13]–[18].

Most ciliates possess two types of nuclei: a transcriptionally active *macronucleus* (soma) and a germline *micronucleus* used for sexual conjugation. After sexual reproduction, the diploid zygotic micronucleus develops into a DNA-rich macronucleus, with each chromosome amplified to a high copy number, with some variation. Non-coding DNA segments (IESs) [19] interrupt most of the ∼30,000 genes in the germline genome of stichotrichous ciliates. Macronuclear development in these species removes intergenic DNA as well as deleting more than 100,000 IESs to allow assembly of the gene segments (MDSs) into functional genes. Fully-assembled genes typically reside on minimalist “gene-sized nanochromosomes” with short telomeres on both ends and almost no non-coding DNA. More surprisingly, the process of gene assembly can even invert and/or permute (switch) segment order, and **scrambled genes** requiring these complicated events may account for 20–30% of all genes in stichotrichs. For example, *actin I*, *telomere-end-binding protein subunit* α, and *DNA polymerase* α, among other genes (e.g. [15], [16], [20]–[22]), contain their germline segments in a permuted order relative to their orthodox (unscrambled) order in the macronucleus. Scrambled segments must be reordered and IESs removed to construct functional, translatable genes in the macronucleus [18].

Homologous recombination between short sequence repeats, called **pointers**, at MDS–IES junctions is involved in gene unscrambling, facilitating both removal of IESs and sorting of MDSs [23]. For example, a DNA sequence present at the junction between MDS *n* and the downstream IES is generally identical to a sequence between MDS *n*+1 and its upstream IES, leading to correct fusion of segment *n* to *n*+1, even over long distances. Despite their presence at all known MDS junctions, pointer repeats are short (average repeat length 4 bp between non-scrambled segments, 9 bp between scrambled segments [24]; current data still agree with these estimates) sometimes with mismatches, implying that pointer recognition alone is insufficient to direct accurate splicing. The repeats may satisfy a structural requirement for DNA splicing, and less of a role in recognition; otherwise incorrectly spliced sequences (results of promiscuous recombination) would dominate. While illegitimate recombination might be a source of new scrambled patterns in the germline [25], macronuclear development retains only the molecules that acquire telomeres at both ends [26], possibly ensuring loss of some promiscuously ordered genes.

The molecular mechanisms of gene unscrambling are not yet understood. Recent studies in several species of ciliates indicate involvement of an RNAi-related mechanism to direct genome-wide DNA rearrangements that may have arisen as a defense against invading genetic agents [27]. A model introduced for *Tetrahymena* and extended to *Paramecium* proposes that small RNAs might function to tag sequences for elimination by a mechanism similar to RNA-mediated gene silencing [28]–[30] (reviewed in [31]). A limitation of this model for stichotrichs is the complexity of events in gene unscrambling. Furthermore, the IESs in these species may be too small to create a specific chromatin structure [32]. Because gene unscrambling requires such an amazing level of precision, current models [29], [33]–[36] with experimental evidence [36] propose that a specific set of templates derived from the old macronucleus may guide assembly, simultaneously repairing imprecise IES excision and directing MDS sorting [33].

To begin to unravel the events of DNA excision and unscrambling during development, we investigated the order of DNA elimination, inversion and reordering in the *actin I* gene in *Stylonychia lemnae*, in a developmental time-course. We chose this locus as our main model system, because it is the simplest scrambled gene whose assembly requires all three types of DNA rearrangements, including deletion, inversion, and permutation, or segment reordering [37]. For comparison, we also extended our analysis to two other scrambled genes in the related ciliate *Oxytricha trifallax* (also called *Sterkiella histriomuscorum*), including its *actin I* ortholog and *telomere-end-binding protein subunit* α (*TEBP*α).

### Structure of the scrambled genes

**Figure 1:**
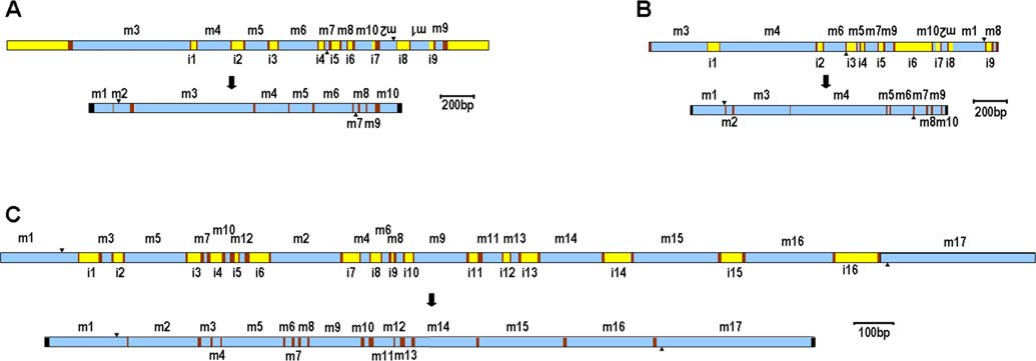
Schematic representation of the micronuclear precursor and resulting macronuclear gene structures (to scale) for (A) *Stylonychia lemnae* (D strain) *actin I*, (B) *Oxytricha trifallax actin I*, and (C) *Oxytricha trifallax TEBP*α. MDSs represented in blue and labeled m (upside down if inverted); IESs (labeled i) and flanking DNA in yellow; pointer sequences, when shown, in maroon. Triangles mark the positions of start (pointing down) and stop codons (pointing up). Black boxes on both ends of the macronuclear structure represent telomeres.

The structures of the micronuclear genes for *actin I* in *Stylonychia lemnae* and its ortholog in *Oxytricha trifallax*, as well as *O. trifallax TEBP*α are shown in Figure 1. The *S. lemnae actin I* gene contains ten MDSs in the scrambled germline order 3 4 5 6 7 8*10*-2-1*9, with segments 1 and 2 inverted. Of the nine IESs, six are conventionally spliced and three, indicated by asterisks, define scrambled junctions. Assembly of *actin I* in *S. lemnae* therefore requires at least six events of conventional DNA deletion (joining segments 3–8 and 1–2), plus insertion of segment 9 between segments 8 and 10 and inversion of segments 1 and 2, as well as their translocation to the 5′ end of the molecule (or inversion of segments 3–10) (see Results section on **DNA Permutation** for more details). Note that the joining of segments 2 to 3, 8 to 9, and 9 to 10 all require permutations. The repeats (pointers) at the boundaries between MDS and IES in these scrambled genes range from 3–19 bp (Table 1–3). In [37] we examined the evolution of the *actin I* gene structure in two geographically isolated strains of *S. lemnae*, which differ significantly in non-coding regions. This study uses only the German strain.

**Table 1:** Characteristics of MDSs, IESs, and pointers of the *S. lemnae actin I* gene.

*MDS m*	*Length* * * of MDS m*	*Pointer sequence between MDS m and m+1*	*IES i*	*between MDSs*	*Length* * * of IES i*
1	85	CACAT	8	1–2	64
2	82	**GAGT(A/C)GTCAAGGCTGGTTT**	7§	10–2̅	15
3	590	TCGTT	1	3–4	23
4	149	AAGA	2	4–5	49
5	117	ATT	3	5–6	46
6	192	TGC	4	6–7	29
7	27	GTTAATTTAT	5	7–8	39
8	28	**GAATCA**	6	8–10	17
9	43	GCCAAGGACAGGTTGAA	9§	1̄–9	29
10	86				

Inverted MDS segments are represented by a bar. MDS numbers *m* are consecutive in the macronucleus, and IES numbers *i* are consecutive in the micronucleus, as in Figure 1. The scrambled micronuclear pointers that link non-consecutive MDSs are boldfaced.

* Lengths of MDSs and IESs exclude pointers.

§ These IESs contain a telomere addition site on one side (beginning of MDS 1 and end of MDS 10) and a scrambled pointer on the other side.

**Table 2 pone-0002330-t002:** Characteristics of MDSs, IESs, and pointers of the *O. trifallax actin I* gene.

*MDS m*	Length* of MDS m	*Pointer sequence between MDS m and m+1*	*IES i*	*between MDSs*	Length* of IES i
1	190	GACCAACAAA	9	1–8	28
2	32	**AAGGCTGGTTTCGC**	8^§^	2̄–1	26
3	227	TCTC	1	3–4	69
4	566	**AGCTCCCAAGTCAA**	2	4–6	29
5	12	**TTATTGCCA**	4	5–7	21
6	31	**TGAGGAAT**	3	6–5	53
7	75	**TGATACTTAAC**	5	7–9	19
8	17	**GGGTTGAATGA**			
9	50	CAAAAAT	6	9–10	215
10	17		7^§^	10–2̄	22

See Table 1 for notation.

**Table 3:** Characteristics of MDSs, IESs, and pointers of the *O. trifallax TEBP*α gene.

*MDS m*	Length* of MDS m	*Pointer sequence between MDS m and m+1*	*IES i*	*between MDSs*	Length* of IES i
1	196	**TGCA(C/A)CAAAGAA**	1	1–3	42
2	293	**TTGTCTTG**	7	2–4	38
3	41	**CACAGACTTGGAG**	2	3–5	6
4	21	**AGAAT(T/C)CACA**	8	4–6	22
5	160	**A(A/C)TCAG**	3	5–7	32
6	33	**AGAAGAATGA**	9	6–8	4
7	18	**TGTTCAAAAC**	4	7–10	26
8	30	AAT	10	8–9	46
9	138	**AGCTTAAG**	11	9–11	20
10	19	**TCAAGTTTT(C/T)T**	5	10–12	9
11	56	**GGTTG(T/C)**	12	11–13	14
12	17	**TCAGCTACTTA**	6	12–2	52
13	28	AAAAGAA	13	13–14	40
14	165	ACAAGAA	14	14–15	64
15	239	TCGTTA	15	15–16	51
16	235	CAGAATT	16	16–17	107
17	397				

See Table 1 for notation.

## Results

### Time-course of DNA deletion during macronuclear development

**Figure 2:**
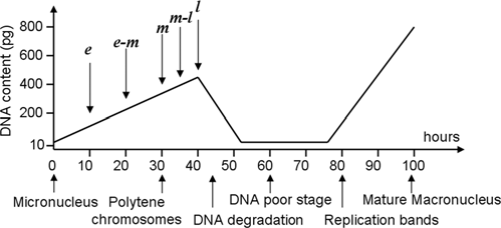
Macronuclear development in *Stylonychia lemnae* (adopted from [49], [52] and consistent with present lab conditions) showing time-points for DNA isolation (*e* = early; *e-m* = early-middle; *m* = middle; *m-l* = middle-late; and *l* = late).

**Figure 3:**
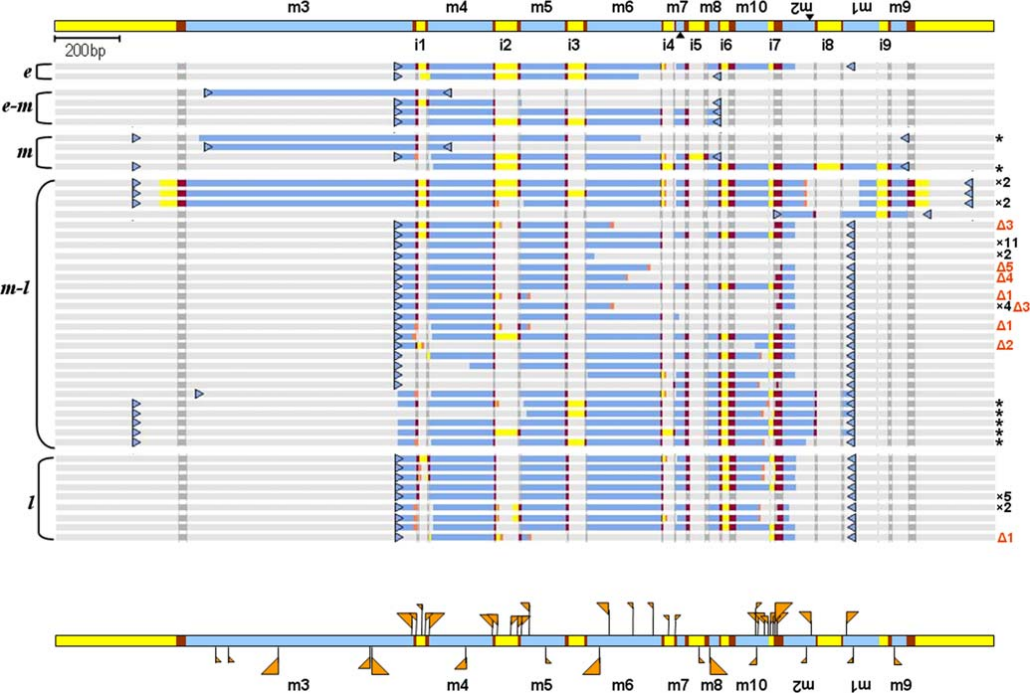
A schematic representation of all partially-processed *S. lemnae actin I* molecules involving conventional DNA deletion junctions (IESs 1–5 and 8) at different stages of development (Figure 2). Regions not covered by amplified molecules are shown in gray (lighter gray for MDS/IES regions and darker gray for authentic pointers). Sequenced MDS regions are blue, IESs and flanking DNA in yellow and authentic pointers in maroon. Cryptic pointers used in some molecules are orange (details in Table S1A and Figure S2). Original PCR primers before nested PCR are shown as blue triangles for each molecule. * indicates sequences with primers in 5′ flanking and 3′ micronuclear gene sequence, described in the text. Numbers after an “x” indicate redundancy of some sequences; orange numbers after a Δ indicate molecules with large deletions (details in Figure S2B). Aligned sequences are provided in Data S1 file “SlActinIConventional.fas”. At the bottom of the figure is a summary of the locations of all cryptic pointers (orange flags) detected in all partially processed molecules. Flags above the molecule represent cryptic pointers flanking aberrant deletions that do not alter micronuclear order. Flags below the molecule represent cryptic pointers found at incorrectly reordered junctions. Flag size is proportional to the number of times each cryptic pointer was observed at a specific location; tallest flags for large deletions (Δ1–5).

To understand the process of IES excision and DNA descrambling, we first examined the population of molecules present during assembly of the *actin I* gene in *S. lemnae*. We PCR amplified DNA from different stages of macronuclear development (called *Anlage*, Figure 2). Anlagen DNA was isolated from partially-synchronized exconjugants, which yield some variation. Five time-points covered a 30-hour period from early polytene chromosome formation to the beginning of DNA degradation (Figure 2). We amplified and sequenced a broad ensemble of partially-rearranged molecules at each time interval using multiple sets of PCR primers (primer sequences are listed in Methods S1; positions are shown in Figure 3 and Figure S1). We then used these “snapshots” of the range of partially-processed molecules to infer the time-course of rearrangement. In addition, we extended this approach to two genes in a related ciliate, *O. trifallax*, to help document the authenticity of partially-rearranged molecules and to avoid being misled by one gene-specific study. The time points used for *O. trifallax* cover a wider range of development stages, from 0-hour (just after mixing of mating types) to 55-hours (DNA poor stage).

To avoid contamination from maternal macronuclear DNA that has not been fully degraded, the sequence and/or orientation of most *S. lemnae* primer-pairs (Methods S1 and Figure S1) cannot amplify the fully-rearranged product, although they can amplify the unprocessed or partially processed micronuclear precursor (Table S1). This strategy enabled us to focus on partially-processed molecules that are incompletely rearranged. For example, the combination of a forward primer from the end of segment 3 (before the first IES) with a reverse primer from inverted segments −2 and/or −1 avoided macronuclear DNA contamination and permitted dense sampling of partially-processed molecules that lacked inversions. In the few cases in which we used primers that could amplify both micronuclear and macronuclear segment order, we recovered similar types of partially-processed molecules, suggesting that most of the observed DNA rearrangements are not artifacts of PCR or primer amplification bias. Furthermore, recent RNAi experiments (See Figures 1b and d in [36]) that disrupted the process of gene unscrambling in *O. trifallax* led to an accumulation of similar partially-processed molecules for two different scrambled genes (*TEBP*α and *DNA polymerase* α), providing independent support for the authenticity of incorrect or partially-rearranged molecules. While this dataset [36] was also generated by PCR, the incorrectly-processed molecules were in greater abundance (permitting PCR cycle number to be small), because the process of rearrangement had been experimentally stalled or halted by depleting specific RNA templates for two independent genes via RNAi. For greater resolution in the present experiments, particularly of scrambled and inverted regions, we amplified shorter intervals, because of the difficulty of capturing long, partially-processed molecules by PCR. We take this difficulty in amplification to indicate either rarity or transience of such molecules, or the presence of nicked or heavily supercoiled DNA [38].

Figure 3 summarizes *all* conventional DNA deletion events involving non-scrambled IESs 1–5 and 8 in *S. lemnae actin I*. IESs are still present in the scrambled regions. All conventional IESs are deleted from most surveyed molecules by the middle-late to late stage of polytene chromosome formation. However, unlike a previous study that used PCR and Southern hybridization to track processing of two simple loci containing just two and three nonscrambled IESs, respectively [39], we detected no specific order of conventional DNA deletion events.

Surprisingly, we found that conventional IES excision can actually be very *imprecise*, with a high level of error at MDS junctions among the molecules surveyed during IES removal (Table S2 and Figure S2). Although the use of primer combinations that amplify the micronuclear-specific segment order may favor molecules that contain errors or are potentially developmentally “stalled”, especially during the middle-late to late stage, we detected similar errors in partially-processed molecules amplified with primers that can amplify both micronuclear and macronuclear segment order, again suggesting that these errors are not experimental artifacts. We also recovered many of the same types of excision errors multiple times from independent experiments and different primers, indicating that the data are unlikely to be PCR artifacts. The data for two genes in *O. trifallax* (Figure S3) contain the same types of partially-processed errors.

All imprecisely joined MDSs in both species were fused at 1–8 nucleotide direct repeats, or *cryptic pointers*, flanking the excised sequence in the precursor molecule, and usually in close proximity to, or within the authentic pointers (details in Figure S2A). The use of cryptic pointers is consistent with a mechanism involving recombination at regions of micro-homology, possibly pausing at such repeats. Errors of both excessive deletion (erosion of coding segments) and insufficient deletion (retention of complete or partial IESs) are present. Assuming that the accuracy of excision at different boundaries is independent, and that the error rates we detect are not highly biased, we can roughly estimate (Table S3) that most molecules are likely to contain at least one incorrect deletion event during development. Since most of these incorrect deletion events disrupt reading frame or protein sequence, in the absence of a selection step or polymerase-mediated proofreading, we can expect that more than three quarters of partially-processed molecules might yield dead-end products, rather than developing into functional macronuclear chromosomes.

Our data (Table S3) do not suggest any correlation between the accuracy of conventional IES deletion and pointer or IES length; *i.e.* the processing of IESs with longer pointers or shorter length is no more robust. However, among cryptic pointers, longer repeats are used more often in spurious rearrangements (Figure S2A and Table S2A). These observations suggest that the events of IES removal may favor longer repeats, although the final accuracy of excision may depend on a step that specifically degrades incorrect products or uses template-directed proofreading [29], [33]–[36].

### DNA Permutation

To address segment reordering, we amplified DNA from different stages of development (Figure 2) using four types of *actin I* primer combinations in *S. lemnae* (Figure S1): (A) forward and reverse primers derived from the 5′ and 3′ flanking regions; (B) a forward primer at the non-scrambled 5′ end of the germline gene and a reverse primer in the 3′ flanking region; (C) a forward primer in the 5′ flanking region and a reverse primer at the 3′ end of the germline gene; and (D) a pair of primers within the micronuclear gene. (A) and (B) yielded products with only conventional IESs removed but no permutations. On the other hand, (C) yielded two types of molecules: The first group (marked * in Figure 3) deleted some conventional IESs, with no segments unscrambled. The second group (Mn101005-20 and Mn101005-14 in Figure S5) deleted ALL conventional IESs and correctly relocated segment 9. Combination (D) produced both un-permuted and permuted rearrangements. The presence of PCR products from (A) with conventional IESs removed suggests that DNA deletion may precede chromosome fragmentation and rearrangement. The lack of rearranged PCR products in combination (B) suggests that fragmentation occurs first at or near the 3′ end (segment 9) prior to descrambling segment 9 (or any other permutations). Breakage at the 5′ end could occur after segment 9 translocation, as supported by the presence of PCR products from (C) that contain both 5′ flanking DNA and segment 9 unscrambled.

**Figure 4:**
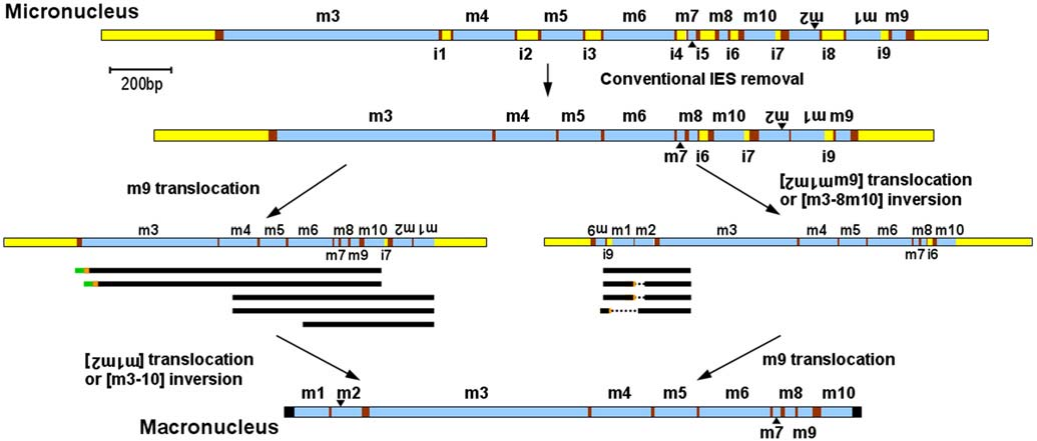
Model for two possible pathways to assemble the *S. lemnae actin I* gene from its precursor form. The data suggest that conventional IES removal precedes unscrambling. Then unscrambling might occur via two alternative pathways that would pass through different transitional stages that are partially-rearranged. We found sequences (represented as black lines under alternative structures, see [Supplementary-material pone.0002330.s005] for details) that support the presence of both types of possible intermediates, suggestingFigure S5 that both pathways may occur, possibly non-deterministically. (Short green lines to the left of black lines represent small duplicated regions of MDS10, and dotted lines represent deleted sequences at cryptic pointers, in orange. Despite mistakes in their processing, these molecules are included in this analysis because they display general features consistent with a possible rearrangement pathway.)

Assembly of a functional *S. lemnae actin I* macronuclear gene requires inversion of segments 1 and 2, coupled with translocation to the 5′ end (or inversion of segments 3–10), plus insertion of segment 9 between 8 and 10, yielding two possible pathways for the permutation events during *actin I* descrambling (Figure 4). We recovered two classes of partially-rearranged molecules, including potential unscrambling intermediates that support both possible pathways (Figure 4). (Some aberrantly rearranged products were also detected that we discuss below.) In the first class of molecules, segment 9 was correctly positioned between 8 and 10, with segments 2 and 1 still in their original inverted orientation at the 3′ end of the molecule. In the second class, either segments 2 and 1 together with segment 9 may be inverted and linked to segment 3 at the 5′ end, or segments 3–10 are inverted and joined to segment 1–2; however, these molecules did not survey processing at the 3′ end, and hence we do not know whether segment 9 is also present between 8 and 10. In addition, three of the four molecules supporting the second pathway also contained cryptic deletions, consistent with the types of errors observed elsewhere. More putative intermediates of the second type were recovered for *O. trifallax actin I* (Figure S4A cases a–c) in an unbiased PCR experiment (primers located in segments 1 and 9) that covered both ends of the molecule, supporting the authenticity of this possible pathway. No examples in *O. trifallax* supported the first pathway (Figure S3A and S4A); however, but this could be due to small sample size. Therefore, both *actin I* descrambling pathways may be evolutionarily feasible, with species-specific biases possible for one or the other pathway.

Some molecules with segment 9 translocations contain another copy of segment 10 at its original micronuclear position (Mn101005-20 and Mn101005-14 in Figure S5), suggesting the possibility of either intermolecular recombination or additional error; however we cannot exclude intra-molecular replication or PCR error to explain the segment 10 duplications. The data for *TEBP*α (Figure S4B) suggest that joining of segments 2–3, 3–4, or 4–5 may occur independently, suggesting multiple pathways for unscrambling this complex gene.

**Figure 5:**
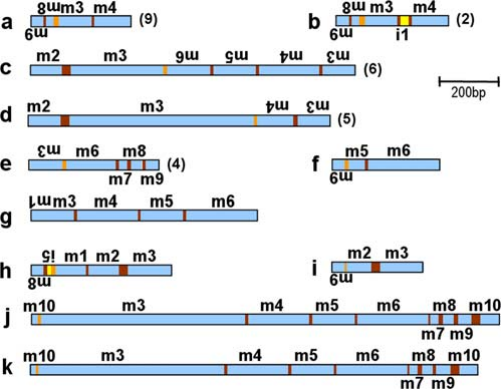
Schematic representation of aberrantly rearranged (mis-unscrambled) *S. lemnae actin I* sequences that do not fit into a productive rearrangement pathway. MDS regions in blue, IESs in yellow and authentic pointers in maroon. Cryptic pointers in aberrant molecules are orange. (The cryptic pointers are provided in the same order in Table S2B, and the sequences of full-length aberrantly rearranged molecules are provided in Figure S5 as well as in Data S1 file “SlActinIPermuted.fas”.) Note that with the exception of case b (recovered twice) and h, all rearranged examples lack IES sequences entirely. In addition, all cryptic pointer repeats in these molecules, with the exception of the left pointer in case h, are present in the macronuclear sequence. Nearly half of the aberrant molecules shown involve illegitimate recombination with a cryptic pointer at the left end of MDS 3 (Figure 3, bottom). This may be partly observer bias, though, because MDS 3 is very long and was surveyed in most experiments. Redundancy of sequences a–e is shown in parentheses.

We also detected various aberrantly rearranged products for *S. lemnae actin I* that do not fit into a possible unscrambling pathway (Figure 5). All but one molecule (case g) contain repeats of 1–10 bp as possible cryptic pointers at their rearranged junctions (details in Table S2B). Aberrant rearrangements can affect both scrambled and non-scrambled regions, displacing pointers at conventional IESs as well as scrambled junctions. Although the cryptic pointers at aberrant junctions are often longer than the non-scrambled pointers they replace (Table S2), they are generally in the same length range as scrambled pointers and usually not longer than the original, displaced scrambled pointer. This suggests that factors other than repeat length drive rearrangement events. Other influences may include the physical proximity of DNA segments in the developing macronucleus. Consistent with this hypothesis, we detect no correlation between the accuracy or robustness of a scrambled junction and the length of its correct pointers (Table S4); however, the junctions between segments 8 and 9 and segments 9 and 10, which are closer to each other in the precursor sequence than the junctions between segments 2 and 3, are much more robust.

Intriguingly, in *S. lemnae* we only recovered molecules with permutations (Figure 5) or large deletions (lines marked Δ in Figure 3; detail in Figure S2B) in the middle-late or late stage of polytene chromosome formation. With just two exceptions (cases b and h in Figure 5), all molecules containing permutations (both correct and aberrant) lack IES sequences entirely, and furthermore contain only correctly excised (or correctly healed) junctions at all nonscrambled IESs. The *O. trifallax actin I* and *TEBP*α data are similar (Fig. S4), although more partially reordered *actin I* molecules in *O. trifallax* retain a small conventional IES. This suggests that the cell executes gross rearrangements, usually by inversion and/or translocation, often after excision and possible repair of all conventional IES junctions, indicating the presence of preferred steps in the gene assembly process. These steps could be associated with the expression onset of specific proteins or factors responsible for different types of reactions during DNA rearrangement [40]. This particular cascade of events both shortens and substantially simplifies the precursor DNA molecule in preparation for the later events of unscrambling, dynamically reducing the complexity of the descrambling problem and opportunity for error. Hence by the onset of more complex events like inversion of segments 2 and 1 or segment 9 translocation in *S. lemnae actin I*, the precursor molecule has already been shortened by 280 bp, the combined lengths of IES 1–5 and 8 plus one copy of each of the flanking pointer sequences that has been consumed. The overall consistency across more than one scrambled gene in two related organisms suggests that this may be a general mechanism for DNA unscrambling. From a theoretical viewpoint, we have called this a “pointer reduction system” [41], and when all correct pointers are eliminated, gene assembly is complete.

## Discussion

We surveyed the population of partially processed DNA molecules from two scrambled genes at different time intervals during macronuclear development in two ciliates, *Stylonychia lemnae* and *Oxytricha trifallax*. Our results revealed several shared characteristics of the gene unscrambling process in *actin I* orthologs in both species (Figure S6) and also in a different scrambled gene (*TEBP*α) in *Oxytricha trifallax*. Firstly, our results showed that conventional IES deletion tends to occur before more complex rearrangement events. Secondly, during the gene unscrambling process, we found evidence to suggest that inversion, translocation or permutation events may occur through multiple parallel pathways, instead of following a deterministic order, although there may be preference for one or the other pathway in different species. Finally, among the surveyed molecules, we observed an unexpectedly high level of error at the boundaries flanking both conventional and scrambled IESs, sometimes associated with abnormal deletion, insertion, or permutation during development. Because most of this error is absent from the mature macronucleus, these observations are consistent with a mechanism that either eliminates or repairs the incorrectly rearranged strands. Together, our results suggest that some events take place in a preferred order, with removal of most nonscrambled IESs first, followed by the key events of DNA inversion and translocation/permutation.

Although an earlier study suggested that the process of IES elimination itself can be highly ordered in *S. lemnae* [39], we did not detect any specific linear order of conventional IES removal in *S. lemnae actin I*. The number of molecules analyzed, however, does not allow a precise test of correlation among IES excision order.

Conventional IES excision in *Stylonychia* and *Oxytricha* may be similar to the phenomena observed in *Tetrahymena* and *Paramecium*, where IESs are most likely marked by small RNAs, leading to modification of local chromatin structure [42], [43] (reviewed in [31]). However, some IESs in *Stylonychia* and *Oxytricha* are smaller than the observed 26–27 nt small RNAs [29], [32], which are also smaller than a nucleosome core particle. This poses a challenge, because small RNAs would be unable to mark such IESs precisely. This could be one source of error in IES excision. IES6 (scrambled between segments 8 and 10) in *S. lemnae actin I* may pose such a problem, as it is 17 bp flanked by a 6 bp pointer on one side and a 17 bp pointer on the other side, and this particular junction is involved in two examples of aberrant rearrangement at 6–8 bp cryptic pointers (cases f and i in Figure 5 and Table S2B).

Because *S. lemnae and O. trifallax* eliminate the majority of their germline DNA (98% and 95%, respectively, with greater micronuclear genome size in *S. lemnae*), and then reorganize a substantial fraction of the DNA that remains, any mistakes of reorganization that yield nonfunctional genes could be fatal. Therefore, these processes must ultimately be very reliable and accurate, which is why the abundance of cryptic pointer junctions in our data was a surprise. Because most pointer sequences are too short to guide accurate IES removal and unscrambling of an entire gene, in part because of redundancy [15], other factors must assist unscrambling. The presence of either selective degradation or a repair mechanism is consistent with our observation that almost all molecules containing permutations have correctly eliminated all conventional IESs (with cases b and h in Figure 5 and cases a, b, and f in Figure S4A the only exceptions). The template-guided model offers a possible solution to these problems [33], [34], [36] with recent experimental support [36]. The proposed templates appear to be complete RNA copies of the DNA molecules present in the maternal macronucleus, appearing transiently in early macronuclear development [36]. The proposed use of maternal templates could simultaneously repair imprecise excision and guide unscrambling [36]. The presence of templates is also consistent with observations in *Paramecium* and *Tetrahymena* of an epigenetic influence of the old macronucleus on DNA rearrangements in the offspring [28], [44]–[47]. This model is compatible with our conclusion that multiple unscrambling pathways may produce functional macronuclear molecules; however, it does not predict the distinct stages that we see during gene unscrambling.

We propose a general mechanism for gene unscrambling involving: marking of DNA sequences for deletion, recombination at adjacent or nearby repeats (that may correctly or incorrectly define segment boundaries), RNA template-guided DNA proofreading of reparable errors or elimination of dead-end molecules containing grossly incorrect junctions, and finally, RNA template-guided recombination at scrambled pointers, yielding correct segment permutation and orientation.

With the exception of one IES in *O. trifallax actin I*, the examined molecules in this study eliminate all conventional IESs before they begin the more complicated steps of inversion and translocation. In *S. lemnae actin I*, only after excision of all conventional IESs do we see evidence for inversion of segments 2 and 1 (or segments 3–10) and translocation of segments 1, 2 and 9. This strategy should increase the system's robustness by reducing opportunity for unscrambling error. It is unknown whether rearrangement of other genes and their scrambled and nonscrambled orthologs in other species proceeds in a similar fashion. Each example may follow a precise pattern influenced by higher-ordered structures of genetic organization [18]. However, our analysis of both orthologous and unrelated genes in two different species consistently suggests that most conventional IESs are eliminated before DNA permutation. Therefore, this may reflect a general mechanism rather than a gene- or species-specific pattern.

The phenomenon of gene unscrambling in stichotrichous ciliates provides a unique model system to examine some of nature's most ornate developmental DNA manipulations. More generally it provides a model system to study epigenetic influences on programmed genome rearrangement [36]. The *Oxytricha trifallax* genome project [48] will ultimately permit a detailed comparison of the *O. trifallax* germline and somatic genomes, revealing thousands of scrambled genes, many as complex as DNA polymerase α [15], and significantly more complex than the *actin I* locus.

## Materials and Methods

### Cells and DNA

*Stylonychia lemnae* were grown in Pringsheim solution and fed daily with *Chlorogonium elongatum* [49]. To achieve conjugation, cells of different mating types (strain D9 and B1) were mixed and the stages of macronuclear development were determined by the size of macronuclear anlage. *Oxytricha trifallax* cells were treated as in [36]. Mating efficiency in both species was over 80% in all experiments and exconjugant survival rate was above 85%. *S. lemnae* anlagen DNA was typically isolated from 5000–10,000 cells at different stages of macronuclear development (Figure 2) and used in PCR amplification as described in [37] and online Methods S1. Due to asynchronous differentiation of individual cells, each time point used in PCR comprises a window of approximately 4–5 hours, which was consistent across experiments in both species.

We collected DNA from conjugating *O. trifallax* cells at 0, 10, 25, 40, 48 and 55 hours after mixing cells of two mating types (strains JRB310 and JRB510). According to a microscopic survey using DAPI stain (not shown), *O. trifallax* conjugating cells reach the peak of the polytenation stage ∼44 hours after mixing and enter the DNA poor stage ∼55 hours after mixing, sharing a similar developmental time scale with *S. lemnae* (polytenation peaks at 42 hours and the DNA poor stage starts ∼52 hours; see Figure 2). Therefore, we label the time points in *O. trifallax* as follows: mixing, early, middle, late-a, late-b and DNA poor stages.

### PCR procedures

DNA sequences flanking the micronuclear *S. lemnae actin I* locus were recovered by UFW PCR [50] as in [25] using primers listed in Methods S1. All *S. lemnae* partially-processed molecules were recovered by nested PCR or a second round of amplification. *O. trifallax* products were recovered in a single conventional PCR, except for one *TEBP*α product amplified with nested IES primers (Methods S1). Negative controls were always performed with water or macronuclear DNA as initial template. See [37] and Methods S1 for more details. Figure S7 provides sample agarose gel images of the PCR time-course for each gene and species.

### DNA cloning and sequencing

*S. lemnae* PCR products were purified (MinElute™ Gel Extraction Kit, Qiagen GmbH, Hilden, Germany) and cloned into pGEM®-T Easy vector (Promega GmbH, Mannheim, Germany). Plasmid DNA was isolated with the QIAprep® Spin Miniprep Kit (Qiagen GmbH, Hilden, Germany) and sequenced by MWG Biotech (Ebersberg, Germany) with T7 and SP6 primers. *O. trifallax* PCR products were purified (QiaQuick PCR Purification Kit, Qiagen, California, USA) and cloned into pCR2.1-topo vector (Invetrogen, California, USA). Plasmid DNA was isolated with the QIAprep® Spin Miniprep Kit (Qiagen, California, USA) and sequenced by Genewiz (New Jersey, USA) or Agencourt (Massachusetts, Germany) with M13 forward and reverse primers.

### Sequence analysis

BioEdit was used for alignment, and computational unscrambling of input sequences was performed with Gene Unscrambler [51].

## Supporting Information

Figure S1 The positions of *S. lemnae actin I* PCR primers and all primer pairs used to amplify molecules with and without permutations. Solid triangles are contained within MDS sequences and empty triangles derive from IES or flanking regions.
(0.16 MB DOC)

Figure S2 Details of the authentic pointers and cryptic pointers found in partially processed *S. lemnae actin I* molecules with inaccurate deletions. A) DNA excision events around each IES. The first line of each set shows the precursor micronuclear sequences at each IES boundary. MDS sequences are in upper case, IES sequences in lower case and authentic pointers in bold upper case. The second line shows correct macronuclear excision products at authentic pointers (shaded red). The remaining set of lines shows the “sloppy” excision events at cryptic pointers (shaded pink). Numbers to the right of each line give the number of times a particular sequence was recovered. For IES 7 and 9, which are unconventional IESs that contain a telomere addition site on one side and a scrambled pointer on the other, the telomere locations are noted. B) Some PCR products contain large deletions that span several MDSs. These molecules can be grouped into five classes (ö1–ö5, also annotated in Figure 3) based on their deletion boundaries. Cryptic pointer-like sequences that flank these large deletions are shaded pink. These molecules are more likely to be dead-end products during erroneous DNA excision than reparable developmental intermediates. Annotation as in previous figures.
(1.33 MB DOC)

Figure S3 A schematic representation of all partially-processed *O. trifallax* (A) *actin I* and (B) *TEBP*α molecules involving conventional DNA deletion junctions at different stages of development (middle = 25 hr, late-a = 40 hr, late-b = 48 hr). MDS regions are blue, IESs yellow and authentic pointers maroon. Cryptic pointers used in some molecules are orange and highlighted by orange triangles. Primer pairs are shown under the schematic micronuclear map in each panel. Solid triangles represent MDS-specific primers; open triangles are IES-specific primers. Triangle direction indicates the strand polarity of the primers. Primer pairs in gray yielded only micronuclear-specific products at all developmental stages. Redundancy of the sequences is shown in parentheses. Aligned sequences are provided in Data S1 files “OtActinIConventional.fas” and “OtTEBPaConventional.fas”, repectively. At the bottom of each panel is a summary of the locations of all cryptic pointers, indicated by orange flags, detected in all partially processed molecules (shown above and in Figure S4). Flags above the molecule represent cryptic pointers associated with aberrant deletions that do not alter micronuclear order. Flags below the molecule represent cryptic pointers found at aberrantly reordered junctions. Flag size is proportional to the number of times a cryptic pointer is observed at a specific location.
(0.11 MB DOC)

Figure S4 Schematic representation of all partially-processed *O. trifallax* (A) *actin I* and (B) *TEBP*α molecules involving permutations. Molecules that do not contain any aberrant deletions or incorrect rearrangement are listed on the left as potential intermediates. Molecules with either aberrant deletions or incorrect permutations are provided on the right. MDS regions are blue, IESs yellow and authentic pointers in maroon. Cryptic pointers are orange and marked by orange triangles. Primer pairs are shown under each micronuclear map. Sold triangles represent MDS-specific primers; open triangles represent IES-specific primers. Triangle direction indicates the strand polarity of the primers. Redundancy of the sequences is shown in parentheses. Figure S3 provides a summary of all cryptic pointers used. All partially permuted sequences in this figure are provided in Data S1 files “OtActinIPermuted.fas” and “OtTEBPaPermuted.fas”.
(0.12 MB DOC)

Figure S5 A dot-plot-like representation of all isolated aberrantly rearranged *S. lemnae actin I* sequences (summarized in Figure 5). The micronuclear structure is represented vertically on the Y-axis. Each molecule is represented horizontally on the X-axis. The matched regions are showed as diagonal lines. The sequences are color-coded in the same way as in Figure 1. The gray lines on the X-axis represent sequences that may be included in the first round of PCR before nested PCR.
(1.78 MB PDF)

Figure S6 Schematic alignment of the orthologous *actin I* macronuclear and micronuclear sequences in *S. lemnae* and *O. trifallax*. Gray areas indicate alignable coding regions.
(0.05 MB DOC)

Figure S7 Representative Ethidium Bromide stained agarose gel images from analysis of PCR reactions that detected partially-processed products for (A) *S. lemnae actin I*, (B) *O. trifallax actin I* and (C) *O. trifallax TEBP*α, at different development stages. In (A), the predominant bands are micronuclear-specific products, and the processed products are indicated by open arrow-heads. M: marker; MAC: macronuclear DNA template control; NT: no template control. Primer combinations are shown beneath each micronuclear map.
(0.30 MB DOC)

Table S1 A summary of PCR results from different primer pairs at different developmental stages for (A) *S. lemnae actin I*, (B) *O. trifallax actin I* and (C) *O. trifallax TEBP*α. The ability of each primer pair to amplify micronuclear (MIC) and macronuclear (MAC) genomic sequences is indicated. The “+” and “−” signs indicate whether partially processed products can be observed among the PCR products, based on either agarose gel analysis or sequencing results.
(0.09 MB DOC)

Table S2 All cryptic pointers found in *S. lemnae actin I* molecules with unusual deletions or aberrant rearrangements. These are grouped based on whether the molecules have (A) deletion without permutation or (B) deletions with aberrant permutation (wrong MDS order). For the cryptic pointers in the first category, (A) lists the deleted IESs (all or partial), the MDS segments that are fused at the cryptic pointer, the number of times that each junction was observed, and the authentic 5′ and 3′ pointers that lie closest to the cryptic pointers in the germline sequence. For the cryptic pointers at aberrantly ordered junctions, (B) lists the observed rearrangements (joining MDS segments *x* to *y*), the number of times that each molecule type was observed, and the authentic pointers that the cryptic pointers replace. In both (A) and (B), underlined nucleotides in the cryptic pointers are contained as a sub-string of the authentic (replaced) pointer, or vice-versa. Double underline is used for overlap when more than one cryptic pointer is a substring of the same actual pointer. Boldfaced nucleotides also overlap in position, *i.e.* the cryptic pointer used all or part of a real pointer on at least one side of the junction. In cases indicated by *, the actual position is a telomere addition site; therefore there are no neighbouring authentic pointers.
(0.08 MB DOC)

Table S3 Robustness analysis of *S. lemnae actin I* nonscrambled pointers (at conventional junctions). The lengths of the pointer and the IES (*i*) between MDS *x* and *y* are listed. N_covered_: number of junctions that are covered in the assayed sequences; N_excised_: number of junctions with an excision event; N_correct_: number of junctions with an excision event at the correct pointer. *IES length excludes pointers. †Assuming that the accuracy of excision at different boundaries is independent, and that the error rates are not highly biased, we can roughly estimate the fraction of molecules that would be correctly-processed at all conventional IES sites by multiplying the values (N_correct_/N_excised_) in the last row of Table S3. Based on this approximation, most (∼78%) molecules might be expected to contain at least one incorrect deletion event during development.
(0.03 MB DOC)

Table S4 Robustness analysis of *S. lemnae actin I* scrambled pointers at permuted junctions. Lengths of scrambled pointers between MDS *x* and *y* are listed. N_processed_: number of junctions with a rearrangement event; N_correct_: number of junctions with a rearrangement event at the correct pointer.
(0.03 MB DOC)

Method S1 (0.05 MB DOC)

Data S1 (0.02 MB ZIP)
