# Supplementary figures and images for: The Pathway to Detangle a Scrambled Gene
Source: PLoS One. 2008 Jun 4;3(6):e2330. doi: 10.1371/journal.pone.0002330 (PMC2394655; doi:10.1371/journal.pone.0002330)

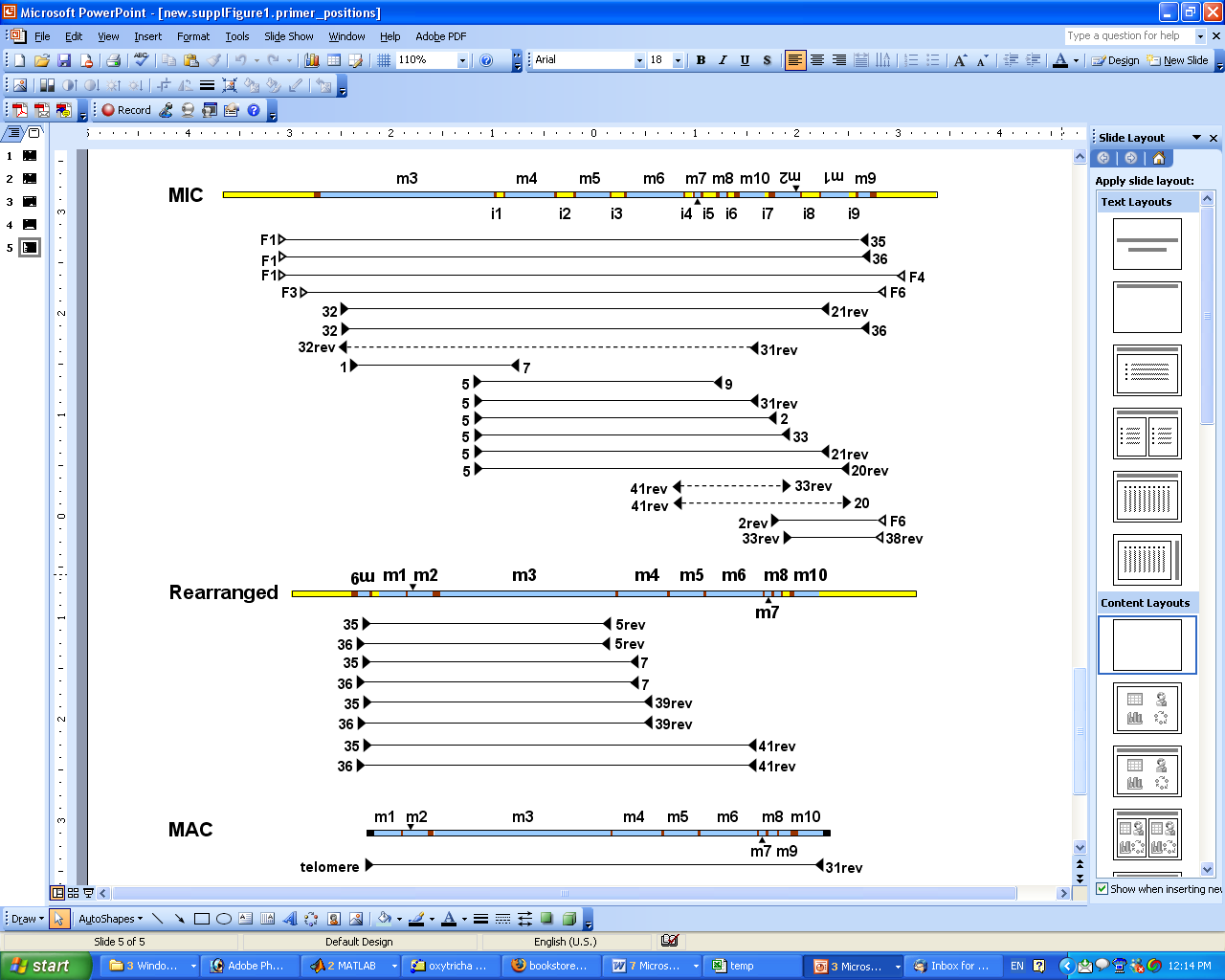

Supplement: Figure S1 — The positions of S. lemnae actin I PCR primers and all primer pairs used to amplify molecules with and without permutations. Solid triangles are contained within MDS sequences and empty triangles derive from IES or flanking regions. (0.16 MB DOC) [file pone.0002330.s001.doc]

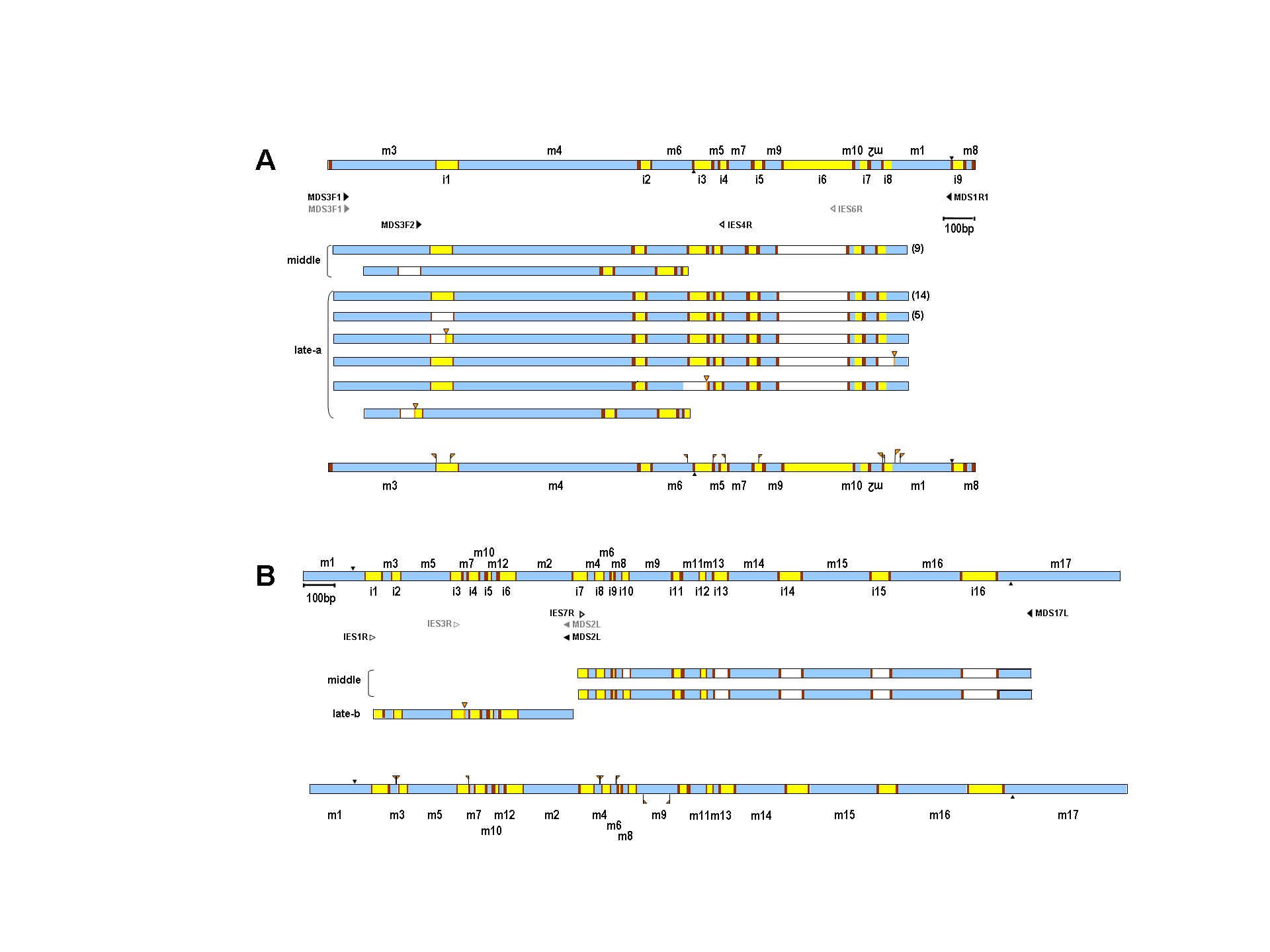

Supplement: Figure S3 — A schematic representation of all partially-processed O. trifallax (A) actin I and (B) TEBPα molecules involving conventional DNA deletion junctions at different stages of development (middle = 25 hr, late-a = 40 hr, late-b = 48 hr). MDS regions are blue, IESs yellow and authentic pointers maroon. Cryptic pointers used in some molecules are orange and highlighted by orange triangles. Primer pairs are shown under the schematic micronuclear map in each panel. Solid triangles represent MDS-specific primers; open triangles are IES-specific primers. Triangle direction indicates the strand polarity of the primers. Primer pairs in gray yielded only micronuclear-specific products at all developmental stages. Redundancy of the sequences is shown in parentheses. Aligned sequences are provided in Data S1 files “OtActinIConventional.fas” and “OtTEBPaConventional.fas”, repectively. At the bottom of each panel is a summary of the locations of all cryptic pointers, indicated by orange flags, detected in all partially processed molecules (shown above and in Figure S4). Flags above the molecule represent cryptic pointers associated with aberrant deletions that do not alter micronuclear order. Flags below the molecule represent cryptic pointers found at aberrantly reordered junctions. Flag size is proportional to the number of times a cryptic pointer is observed at a specific location. (0.11 MB DOC) [file pone.0002330.s003.doc]

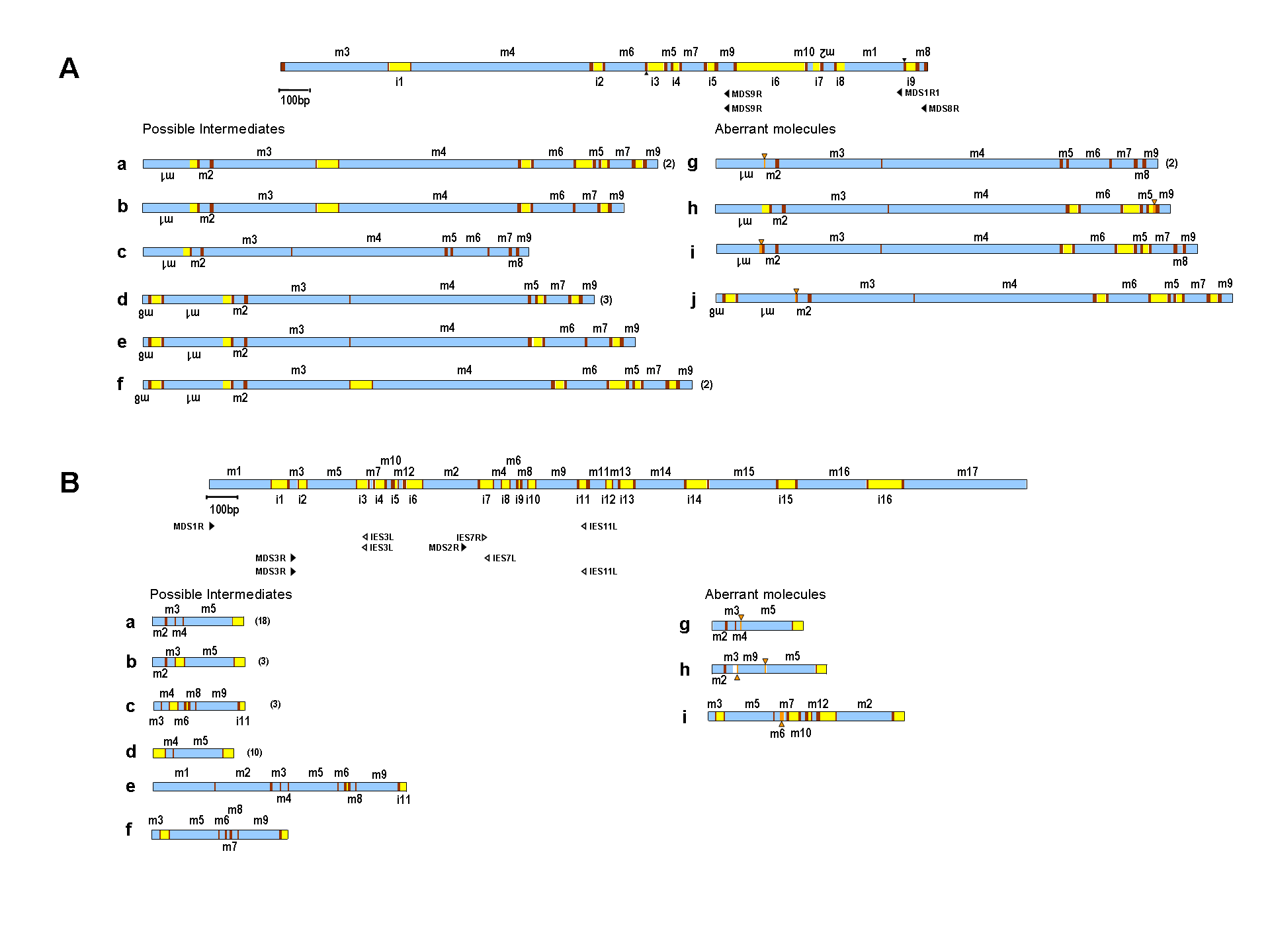

Supplement: Figure S4 — Schematic representation of all partially-processed O. trifallax (A) actin I and (B) TEBPα molecules involving permutations. Molecules that do not contain any aberrant deletions or incorrect rearrangement are listed on the left as potential intermediates. Molecules with either aberrant deletions or incorrect permutations are provided on the right. MDS regions are blue, IESs yellow and authentic pointers in maroon. Cryptic pointers are orange and marked by orange triangles. Primer pairs are shown under each micronuclear map. Sold triangles represent MDS-specific primers; open triangles represent IES-specific primers. Triangle direction indicates the strand polarity of the primers. Redundancy of the sequences is shown in parentheses. Figure S3 provides a summary of all cryptic pointers used. All partially permuted sequences in this figure are provided in Data S1 files “OtActinIPermuted.fas” and “OtTEBPaPermuted.fas”. (0.12 MB DOC) [file pone.0002330.s004.doc]

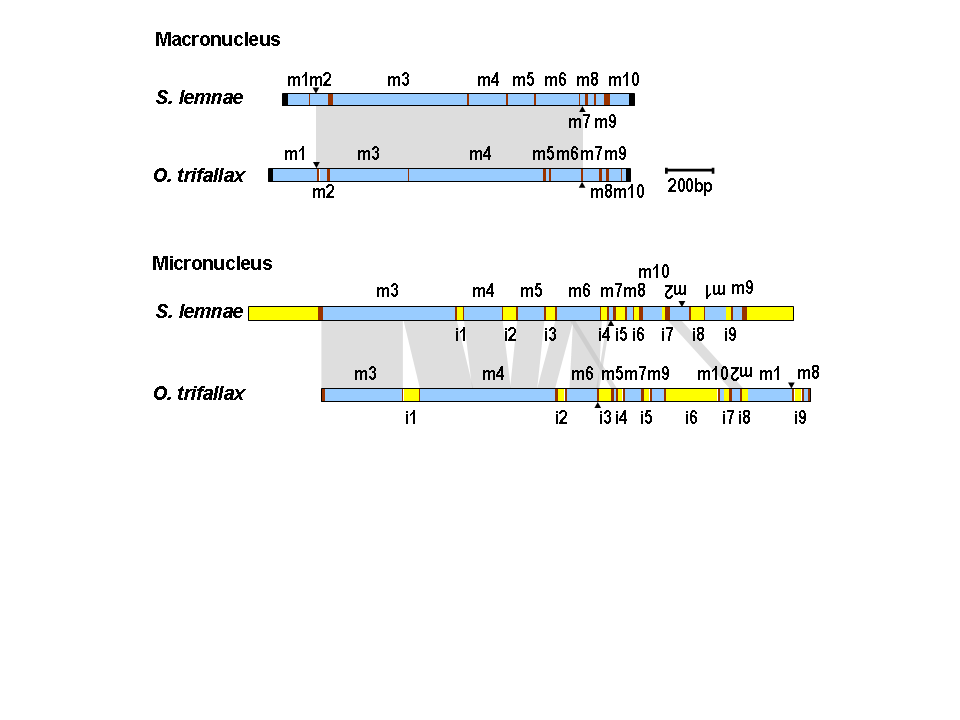

Supplement: Figure S6 — Schematic alignment of the orthologous actin I macronuclear and micronuclear sequences in S. lemnae and O. trifallax. Gray areas indicate alignable coding regions. (0.05 MB DOC) [file pone.0002330.s006.doc]

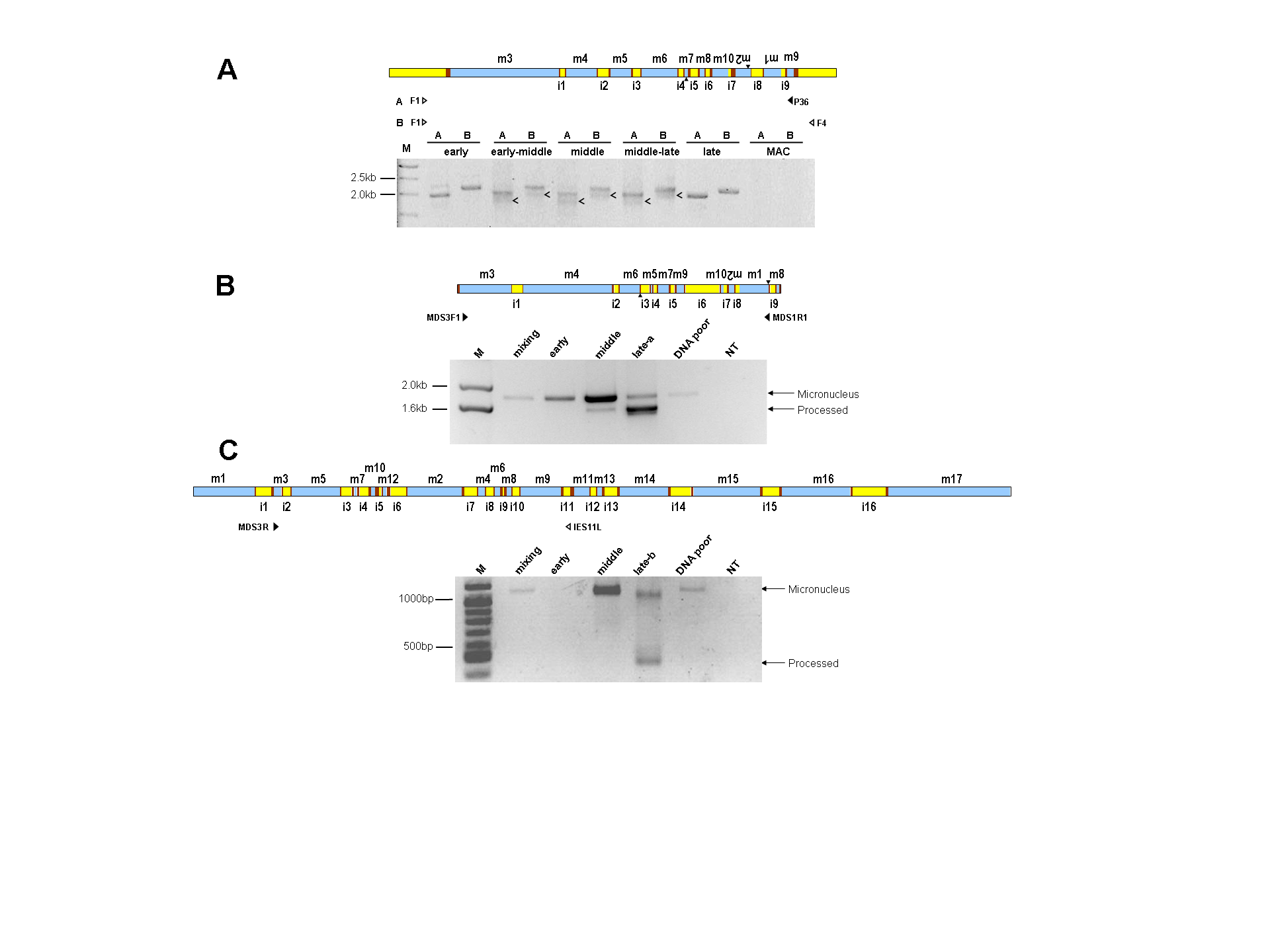

Supplement: Figure S7 — Representative Ethidium Bromide stained agarose gel images from analysis of PCR reactions that detected partially-processed products for (A) S. lemnae actin I, (B) O. trifallax actin I and (C) O. trifallax TEBPα, at different development stages. In (A), the predominant bands are micronuclear-specific products, and the processed products are indicated by open arrow-heads. M: marker; MAC: macronuclear DNA template control; NT: no template control. Primer combinations are shown beneath each micronuclear map. (0.30 MB DOC) [file pone.0002330.s007.doc]
