# Supplementary material for: The Pathway to Detangle a Scrambled Gene
Source: PLoS One. 2008 Jun 4;3(6):e2330. doi: 10.1371/journal.pone.0002330 (PMC2394655; doi:10.1371/journal.pone.0002330)
Supplement: Figure S2 — Details of the authentic pointers and cryptic pointers found in partially processed S. lemnae actin I molecules with inaccurate deletions. A) DNA excision events around each IES. The first line of each set shows the precursor micronuclear sequences at each IES boundary. MDS sequences are in upper case, IES sequences in lower case and authentic pointers in bold upper case. The second line shows correct macronuclear excision products at authentic pointers (shaded red). The remaining set of lines shows the “sloppy” excision events at cryptic pointers (shaded pink). Numbers to the right of each line give the number of times a particular sequence was recovered. For IES 7 and 9, which are unconventional IESs that contain a telomere addition site on one side and a scrambled pointer on the other, the telomere locations are noted. B) Some PCR products contain large deletions that span several MDSs. These molecules can be grouped into five classes (ö1–ö5, also annotated in Figure 3) based on their deletion boundaries. Cryptic pointer-like sequences that flank these large deletions are shaded pink. These molecules are more likely to be dead-end products during erroneous DNA excision than reparable developmental intermediates. Annotation as in previous figures. (1.33 MB DOC) [file pone.0002330.s002.doc]

**A.**


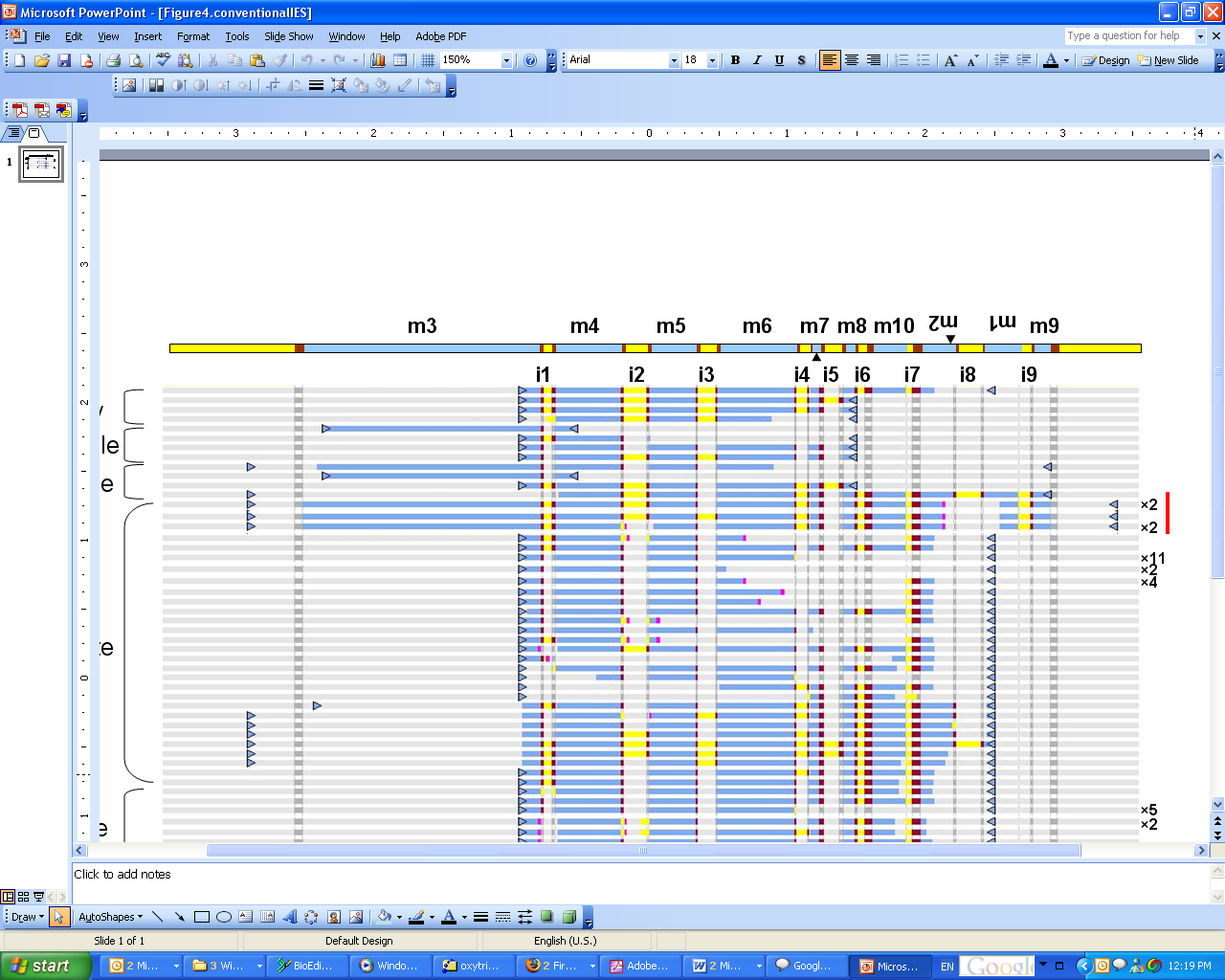


**IES1**

AGCTTTGCT**TCGTT**caatcattattaaatcaaattaa**TCGTT**GCTTTGGACTACGAATC 12

AGCTTTGCT**TCGTT**----------------------------GCTTTGGACTACGAATC 36

AGCTTTG-----------------------------------------GACTACGAATC 5

AGCTTTGCT**T**------------------------------------TGGACTACGAATC 4

AGCTTTGCT**TCGTT**caatc--------------------**GTT**GCTTTGGACTACGAATC 1

AGCTTTGCT**TCGTT**caa-------------------a**TCGTT**GCTTTGGACTACGAATC 1

**IES2**

CGG**AAGA**tttcttcctttaatttattaatagaaaataaatccaatattctaaattt**AAGA**GAGCTCTCCTCAATCC 11

CGG**AAGA**-----------------------------------------------------GAGCTCCCCTCAATCC 40 CGG**AAGA**tttcttcctttaattt---------------------------------**AAGA**GAGCTCTCCTCAATCC 4

CGG**AAGA**tttcttcc---------------------------aatattctaaattt**AAGA**GAGCTCTCCCCAATCC 3

CGG**AAGA**ttacatcct------------------------------------------------------CAATCC 3

**IES3**

GAAGGT**ATT**cactaatcatccctactagctatgctaattgattgtggtataatg**ATT**GGTGAGAGACTC 10

GAAGGT**ATT**------------------------------------------------GGTGAGAGACTC 50

**IES4**

AAAG**TGC**ttactaataatagcatttaattatataaa**TGC**ATCTGAGCAAT 6

AAAG**TGC**--------------------------------ATCTGAGCAAT 18

AAAG**TGC**ttactaataatagcat------------------CTGAGCAAT 1

**IES5**

ATG**GTTAATTTAT**cattagccataaatctaatattttttcatctctctaata**GTTAATTTAT**GCTGAAT 4

ATG**GTTAATTTAT**-------------------------------------------------GCTGAAT 29

**IES6 *(scrambled)***

GGAGCCCTCC**GAATCA**tatcatataataatgtt**GCCAAGGACAGGTTGAA**TGAGGTTGGTGA

GGAGCCCTCC**GAATCA** **-----------------**TGAGGTTGGTGA

**IES7 *(scrambled)*** telomere

TTCCACTTATTTATACTAAATTCCATCAAATCtactaaatttatgtt**AAACCAGCCTTGACGACTC**CGGA 21

TTCCACTTATTTATACTAAATTCCATCAAATC **-------------------**CGGA

TTCCA------------------------------------------------**GCCTTGACGACTC**CGGA 4

TTCCACTTATTTAT------------------------------gtt**AAACCAGCCTTGACGACTC**CGGA 3

TTCCACTTAT----------------------------------gtt**AAACCAGCCTTGACGACTC**CGGA 1

TTCCACTTATTTAtactaaattccatcaaa--------------------**CCAGCCTTGACGACTC**CGGA 1

TTCCACTTATTTATACTAAATTCCATCAAACC--------------------**AGCCTTGACGACTC**CGGA 1

**IES8^*^**

TTCTAGAGATTTAATATATTATTTATCAAG**ATATG**t(61bp)ta**ATATG**CCCTTACTTAAATATCCTTGCTAATTAATATAAATTGAGATATCTCTGACAA 2

TTCTAGAGATTTAATATATTATTTATCAAG**ATATG**-(61bp)-------CCCTTACTTAAATATCCTTGCTAATXXXXXXXXXXXXXXXXXXXXXXXXXXX 5

TTCTAGAGAT--------------------------(61bp)------------------------------------------------ATCTCTGACAA 5

***Note: MDS 1-2 and the pointer CATAT are on the opposite strand.**

**IES9 *(scrambled)*** telomere

CATCTAATACTCTCTATAgaaattatttaattggtatggatgtcttta**GAATCA**GTGCCTCTTCAATACT

CATCTAATACTCTCTATA **------**GTGCCTCTTCAATACT


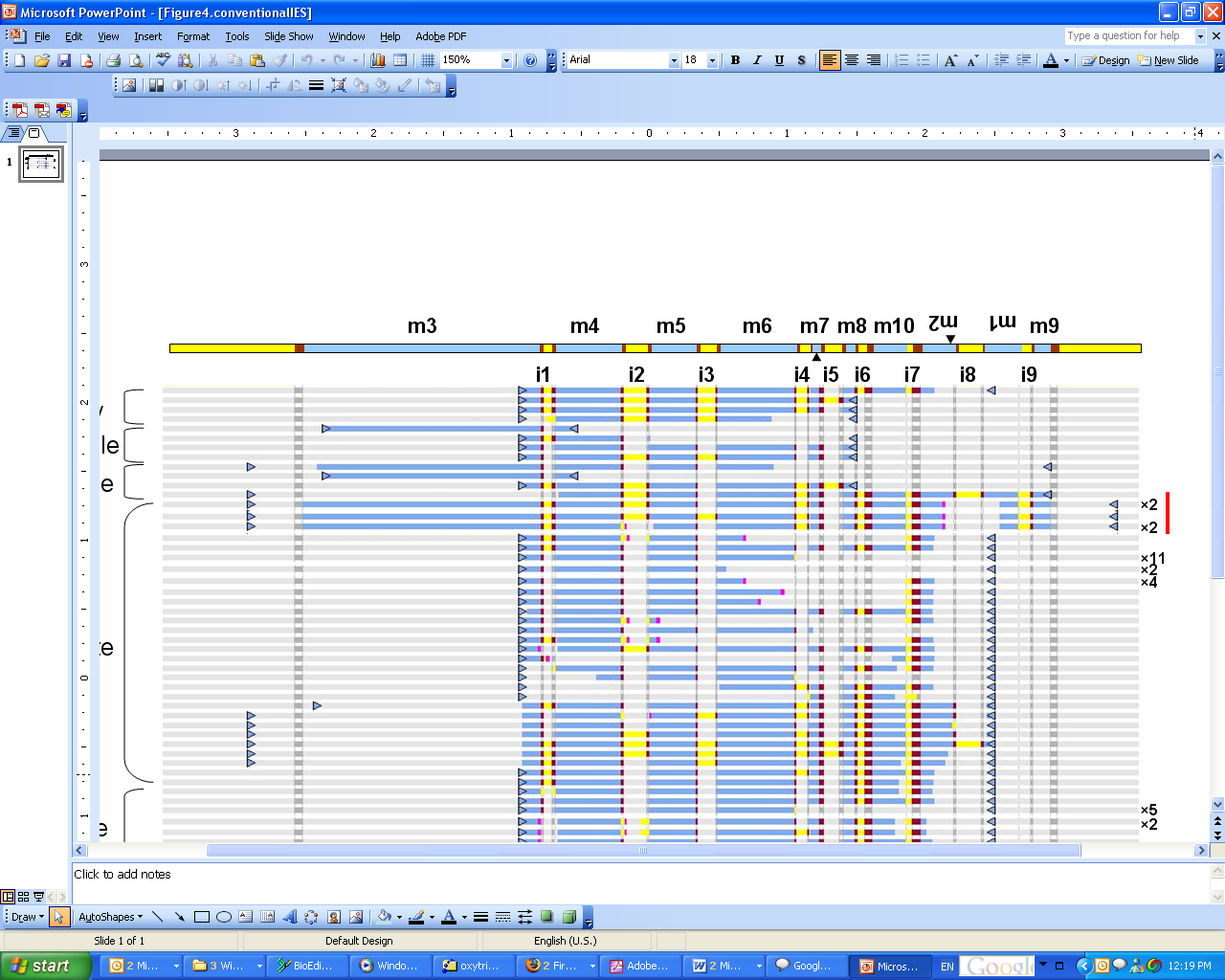


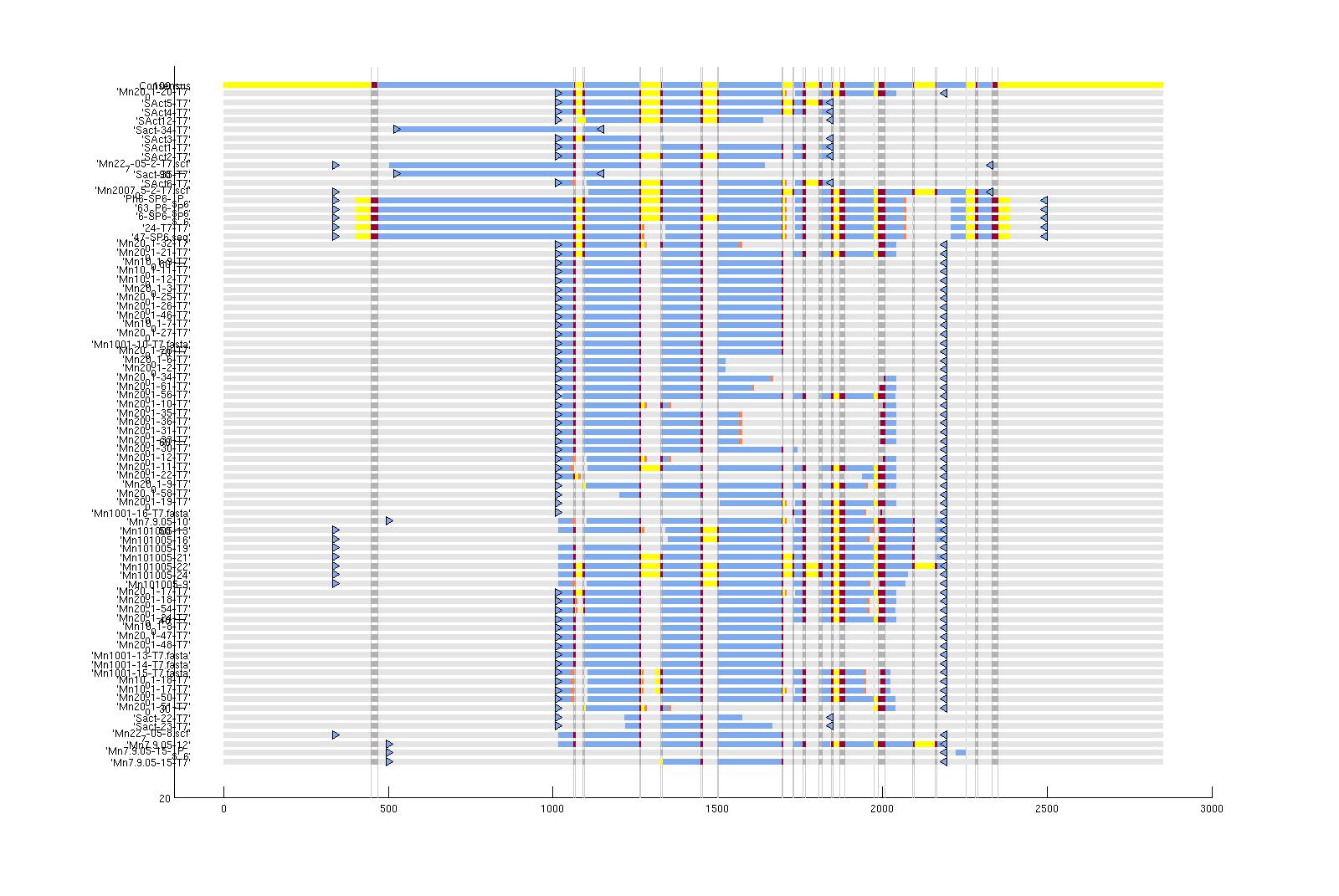

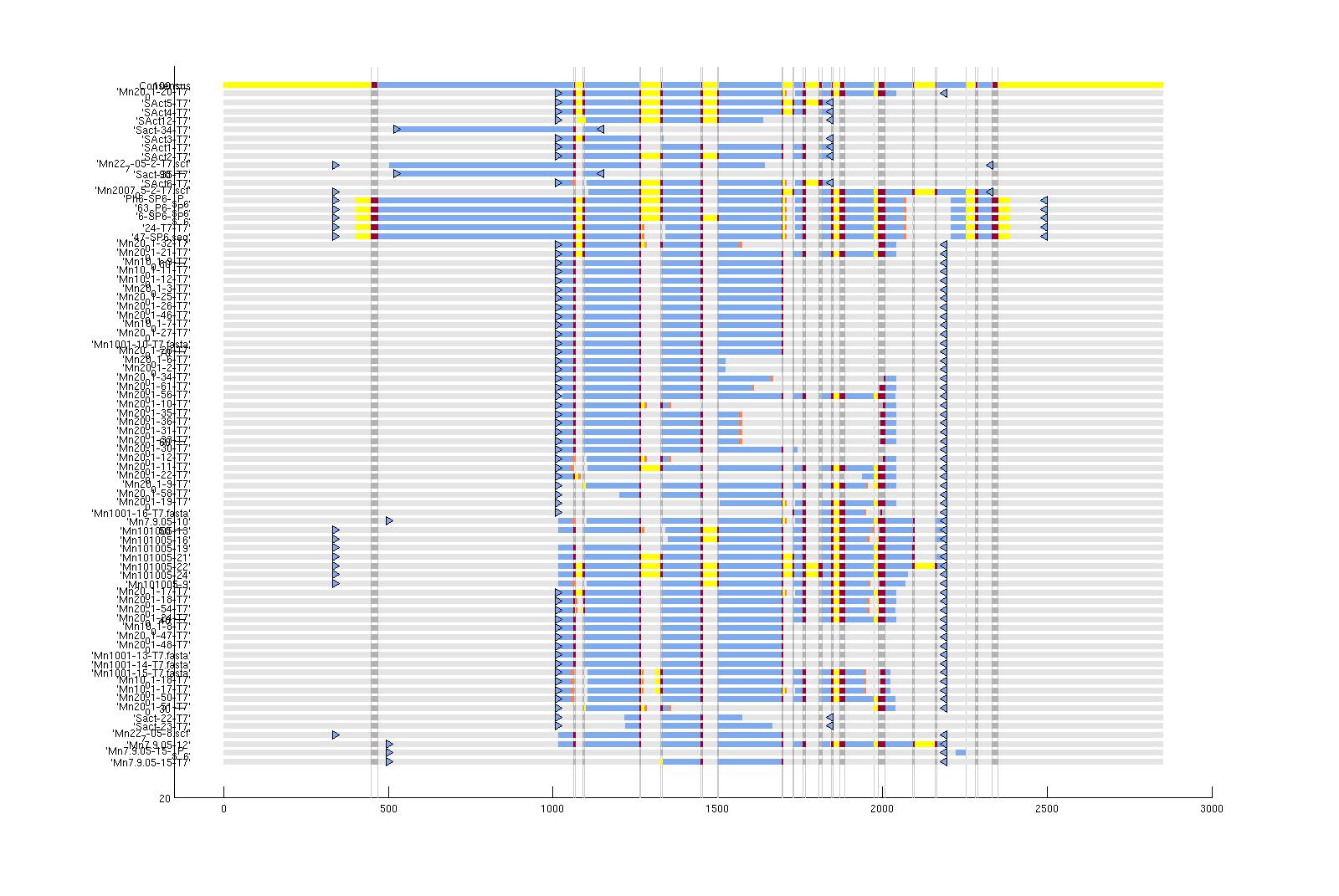

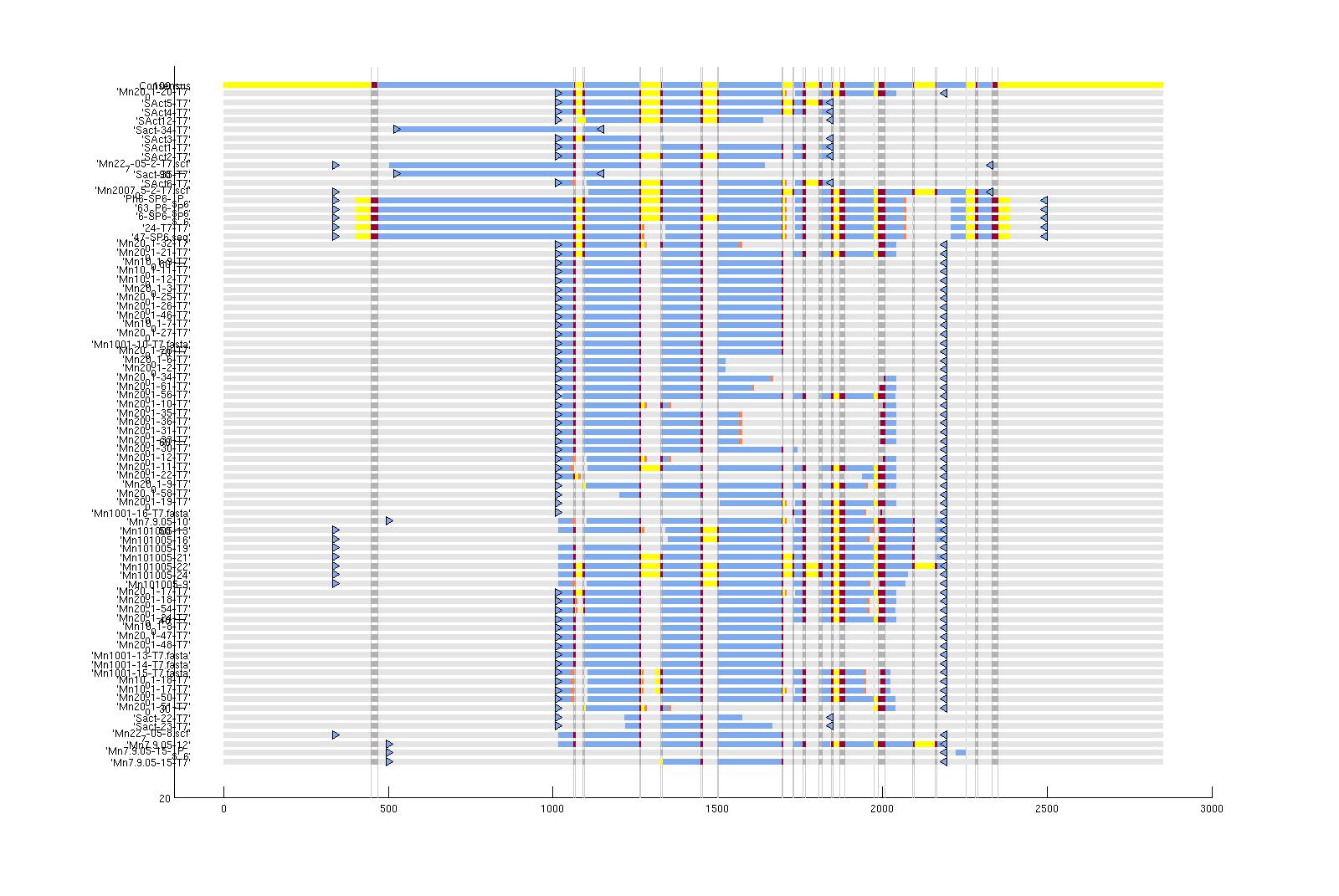

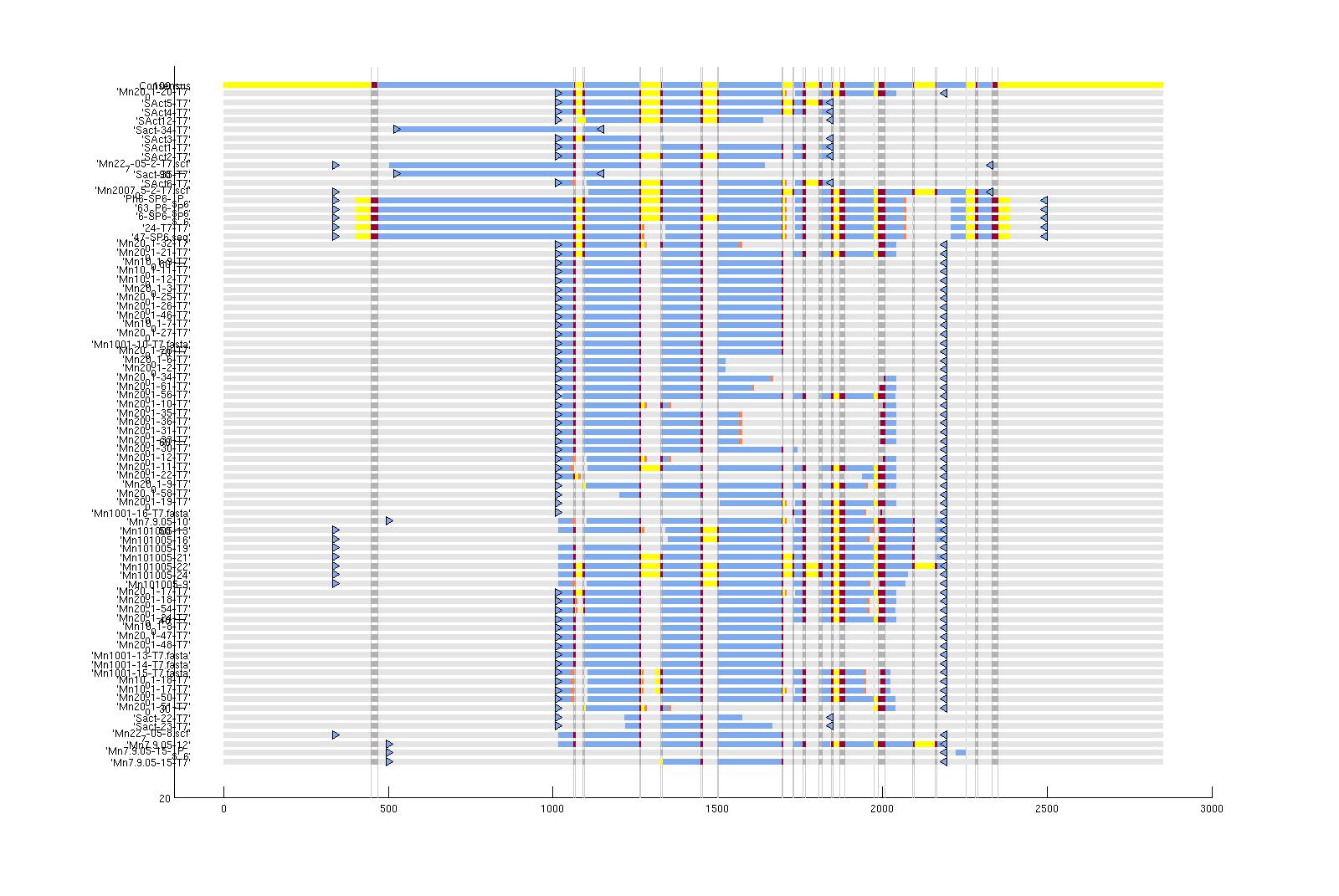

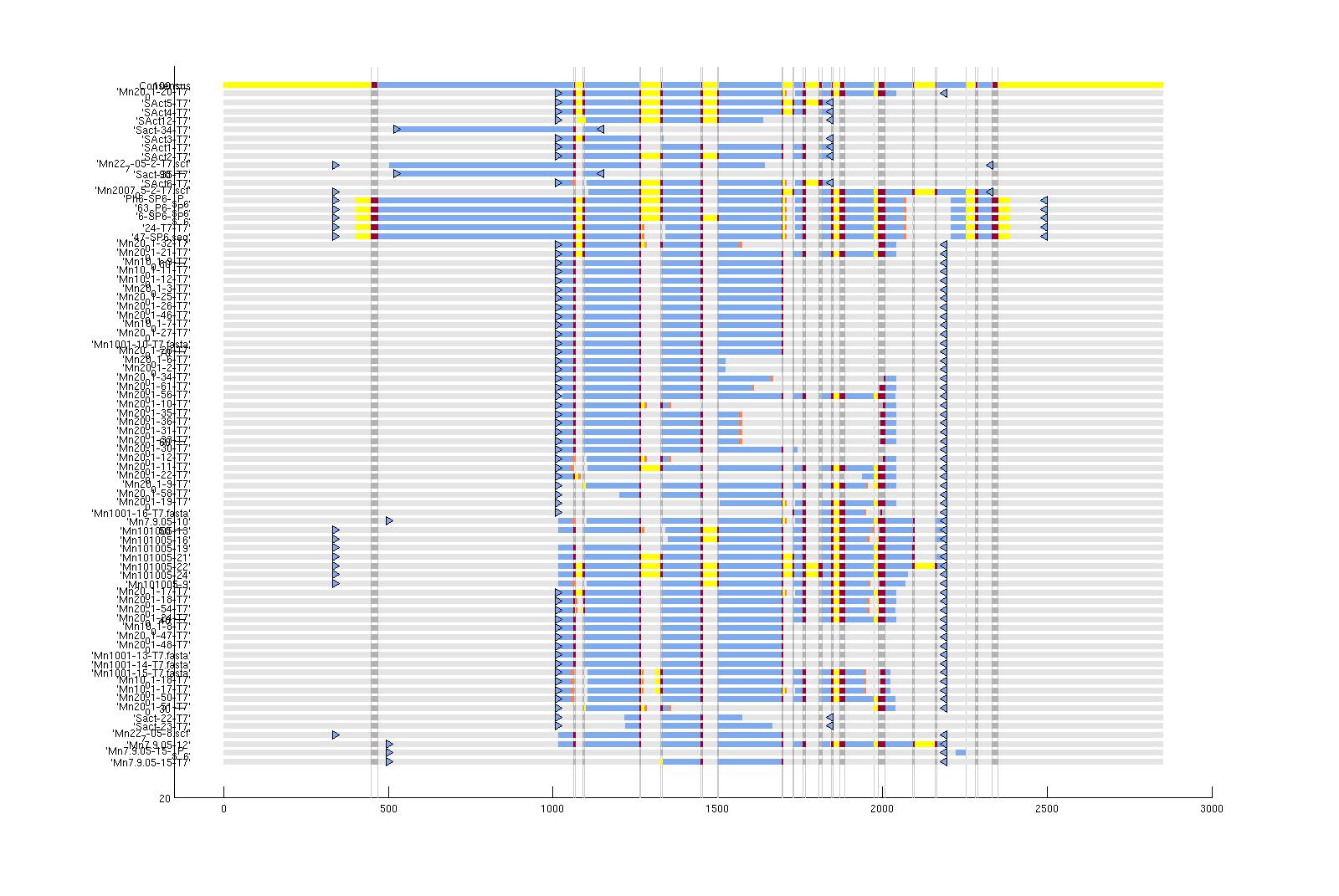

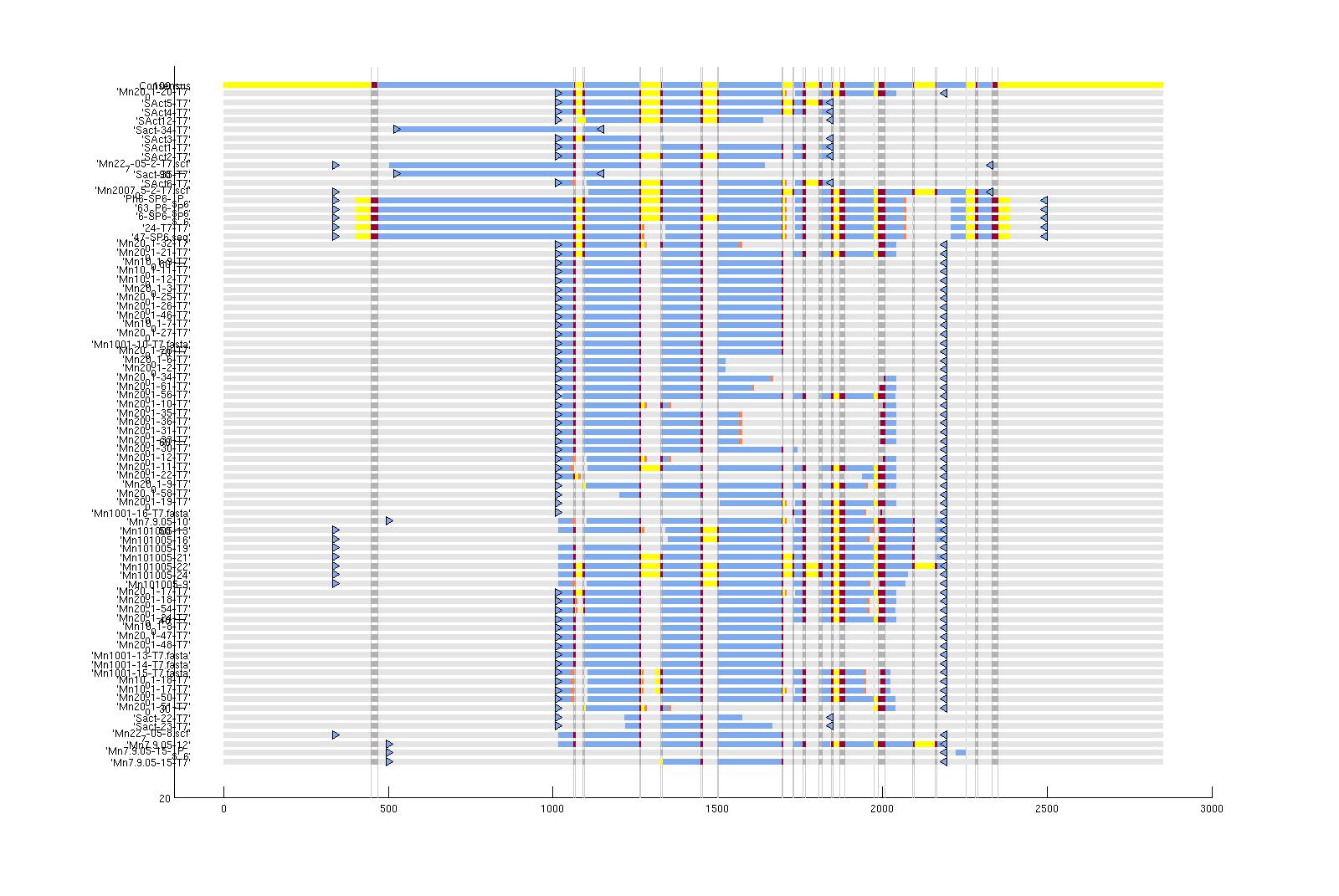

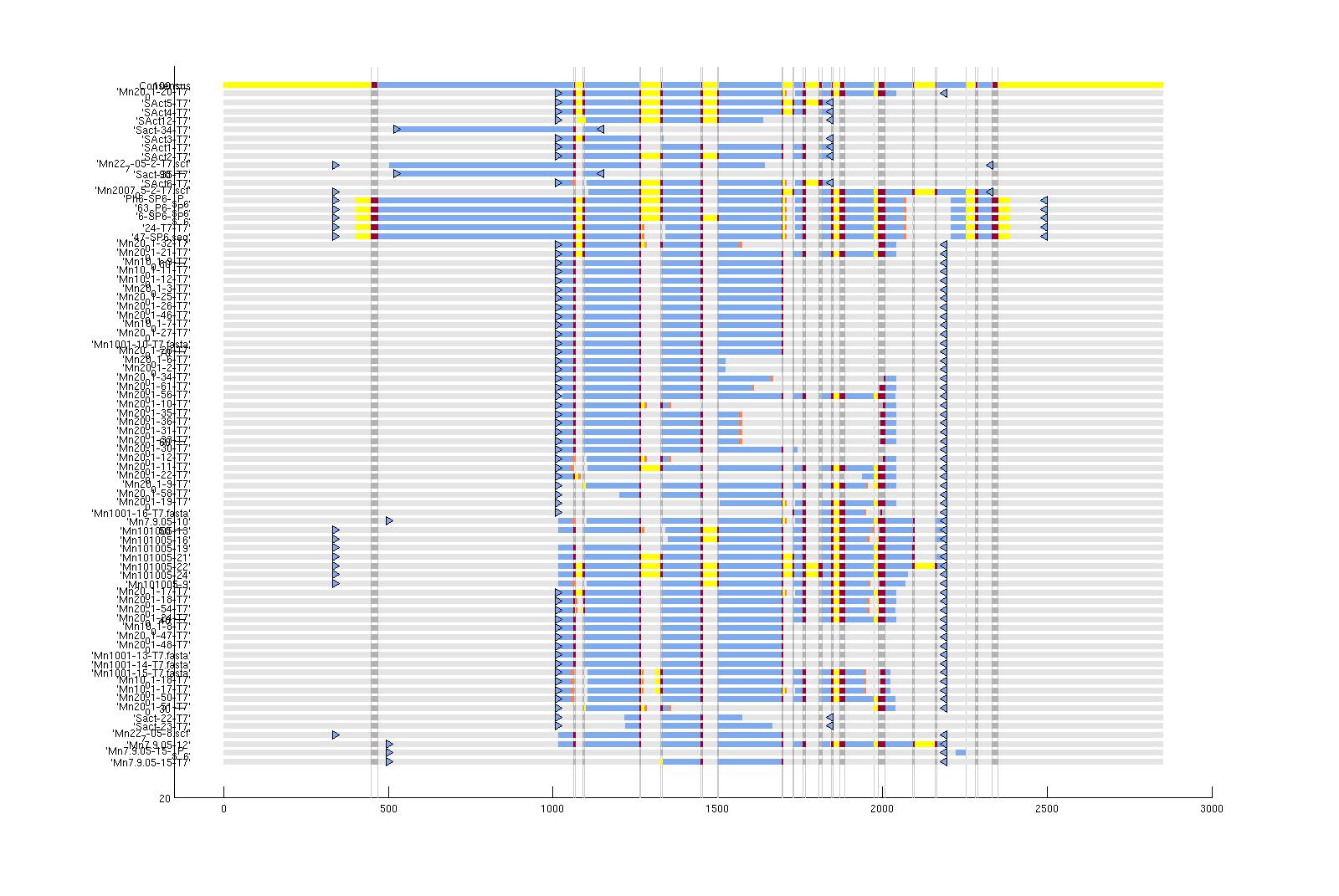

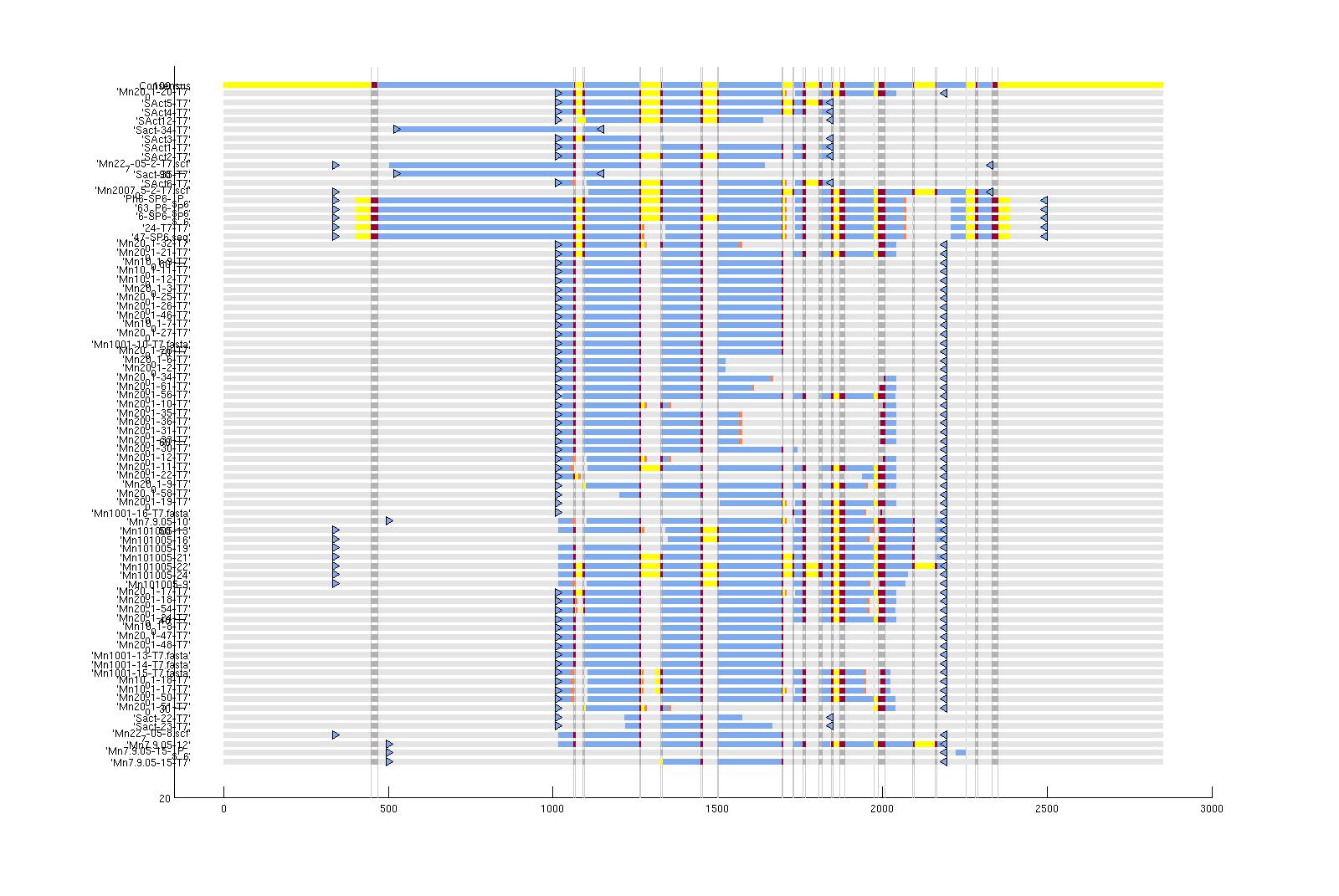

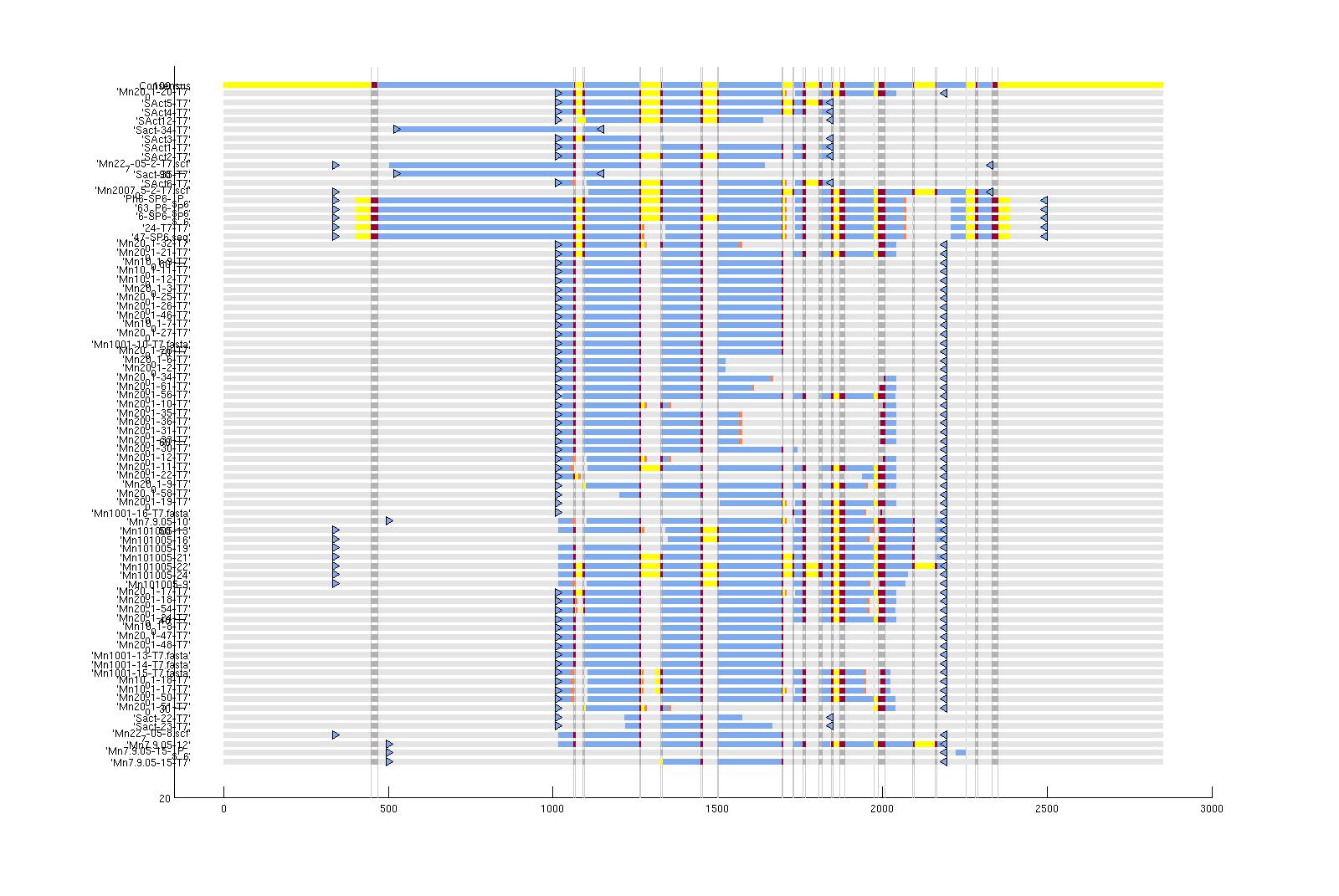

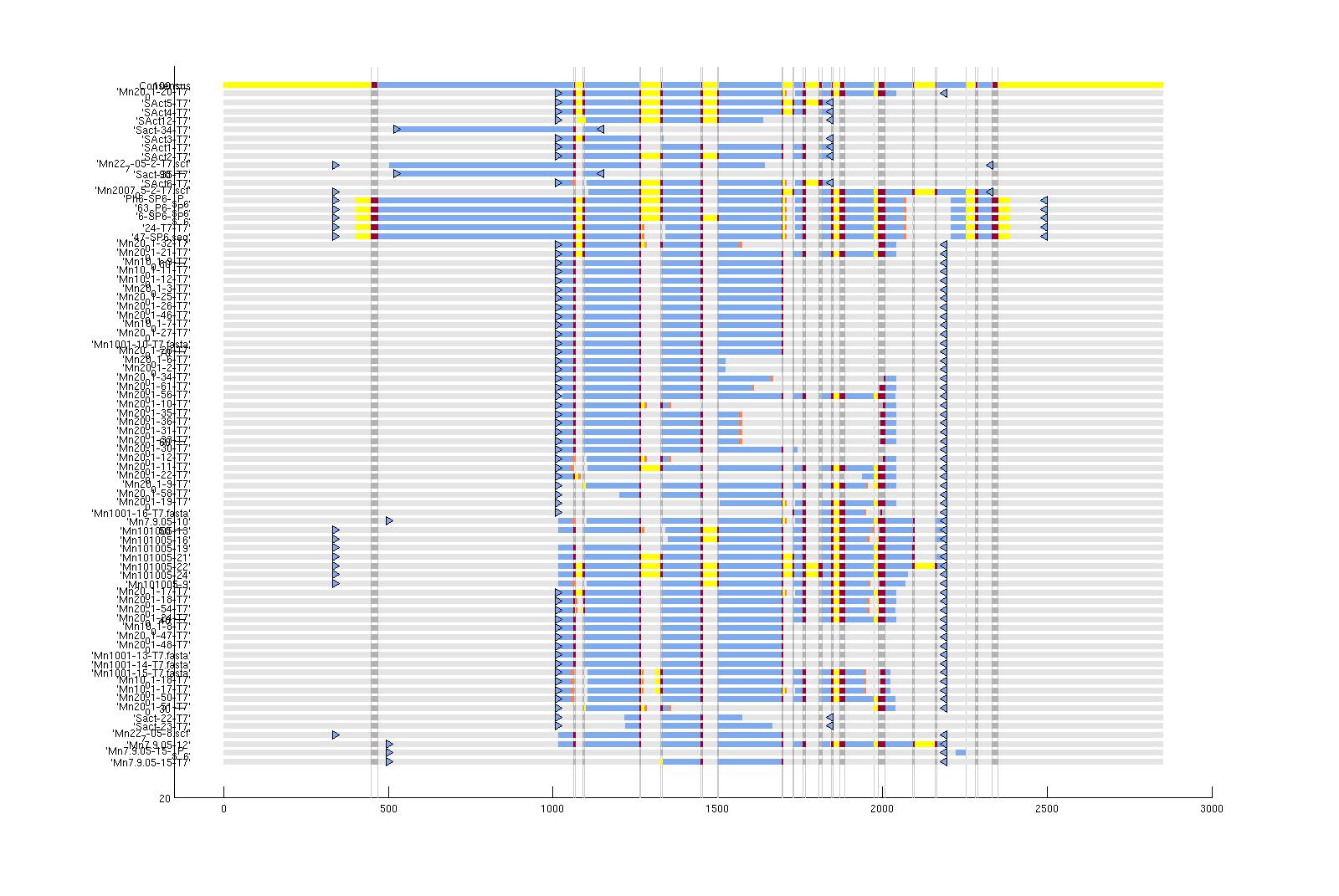

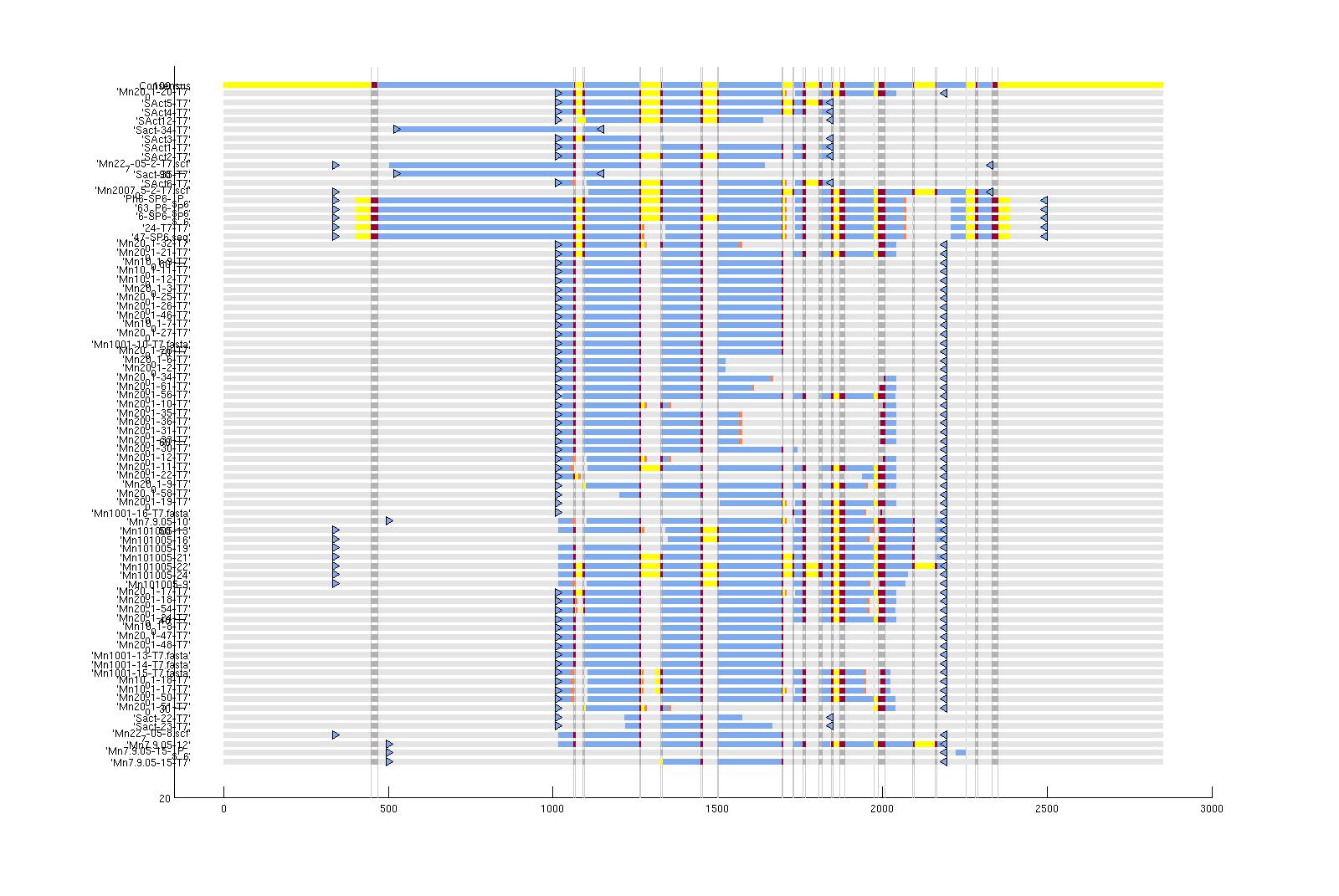

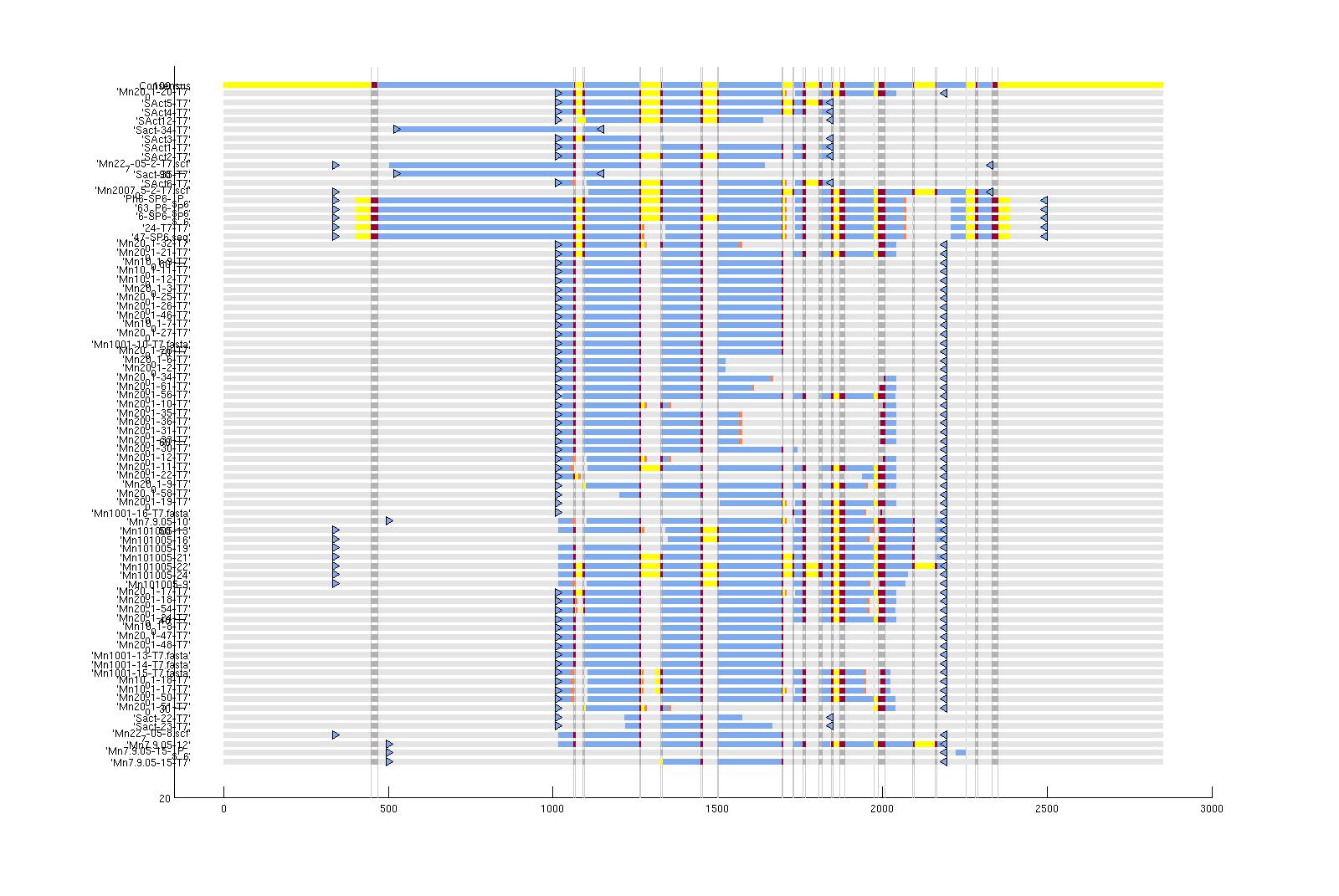


**B.**

**Δ1**

**Δ2**

**Δ3**

**Δ4**

**Δ5**

**Δ1**

**m5**

CCTCAATCCAAGACTTGACCTACAACTC **…… i3-i7 ……** tt**AAACCAGCCTTGACGACTC**CGGATCCATT

CCTCAATCCAAGACTTGAC--------- ----------------**GACTC**CGGATCCATT 3

**Δ2**

**i1**

**m10**

tcaatcattattaaatcaaattaa **…… m4-i6 ……** TTCATAAATATTAAATGCAAATTCCACTTAT

tcaatcattattaaat-------- ----------------GCAAATTCCACTTAT 1

**Δ3**

**m6**

AAAGTTATTGCCAGCCCAGACAGAAGATTCG **… i4-i7 …** tt**AAACCAGCCTTGACGACT**CCGGATCCATT

AAAGTTATTGCCAGCC--------------- -----------**TTGACGACT**CCGGATCCATT 5

**Δ4**

**m6**

AGGTTCCACCCTTACATCTCTCT **…… i4-i7 ……** tt**AAACCAGCCTTGACGACTC**CGGATCCATT

AGGTTCCA--------------- --------GCCTT**GACGACT**CCGGATCCATT 1

**Δ5**

**m6**

GAAGACTATGACGAGCACGGAGCCCAAA **…… i4-i7 ……** tt**AAACCAGCCTTGACGACTC**CGGATCCATT

GAAGACTATGACGA-------------- ------------------**CT**CCGGATCCATT 1
