# Supplementary material for: The Pathway to Detangle a Scrambled Gene
Source: PLoS One. 2008 Jun 4;3(6):e2330. doi: 10.1371/journal.pone.0002330 (PMC2394655; doi:10.1371/journal.pone.0002330)

Case a:

Mn23606-7T7

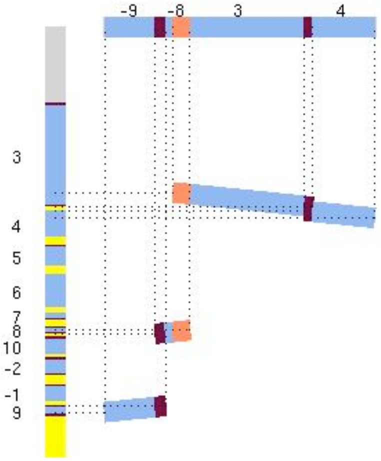

Mn23606-8T7

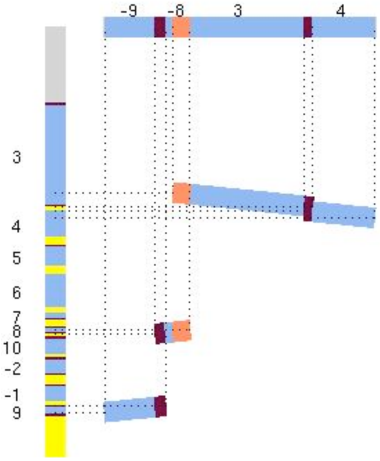

Mn23606-10T7

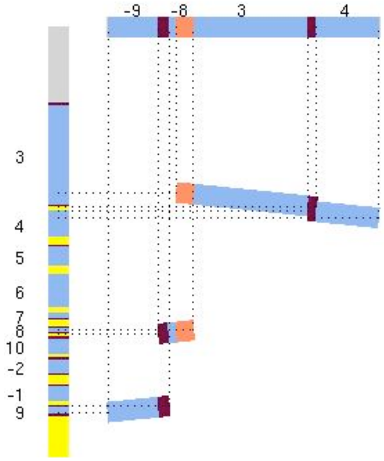

Mn23606-11T7

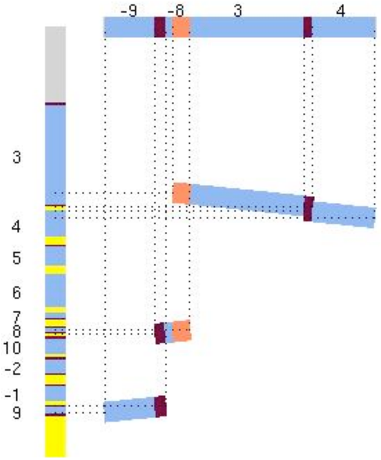

Mn23606-6T7

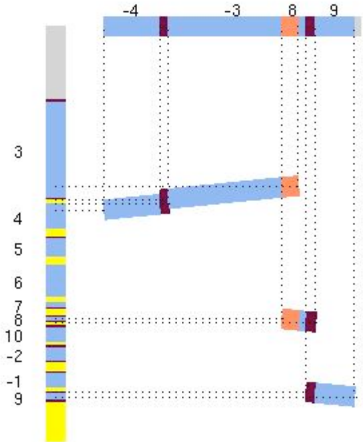

Mn23606-14T7

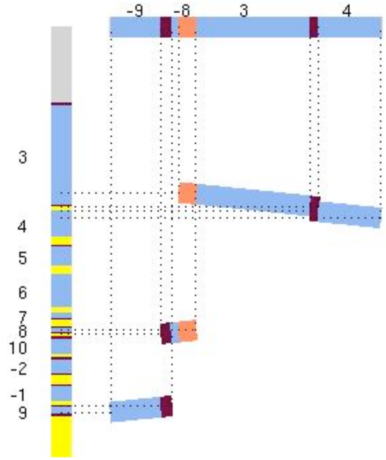

Mn23606-15T7

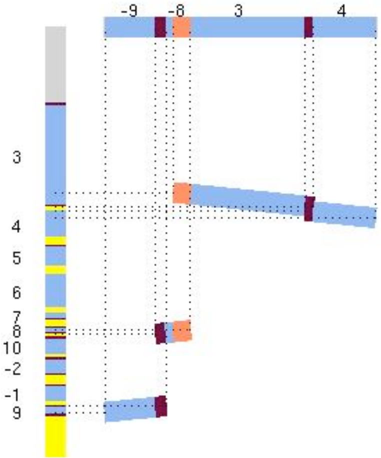

Mn23606-23T7

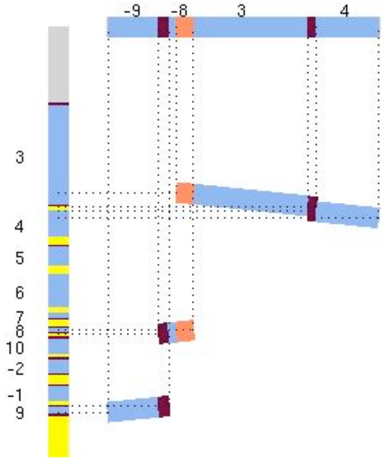

Mn23606-24T7

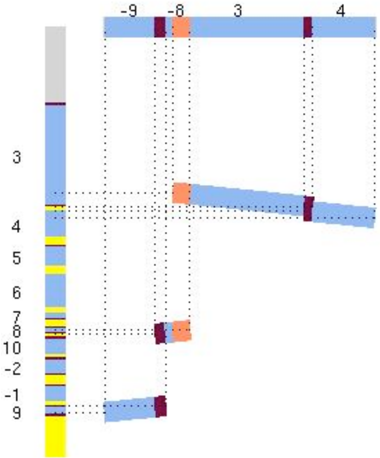

Case b:

Mn23606-12T7

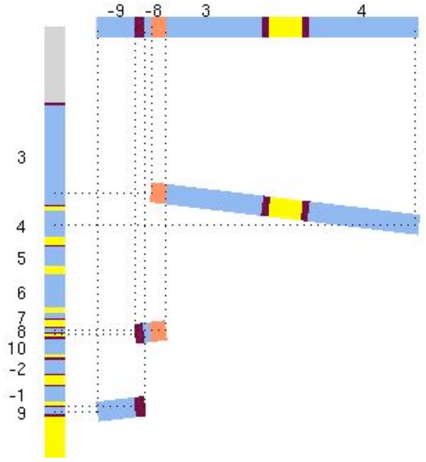

Mn23606-21T7

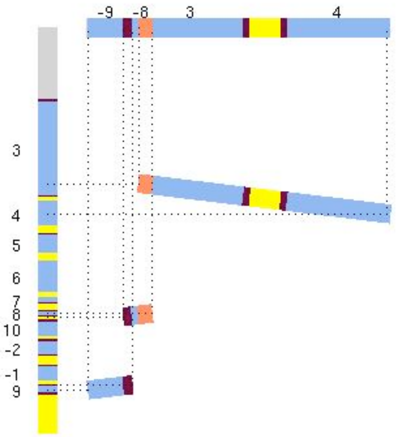

Case c:

Mn2001-14

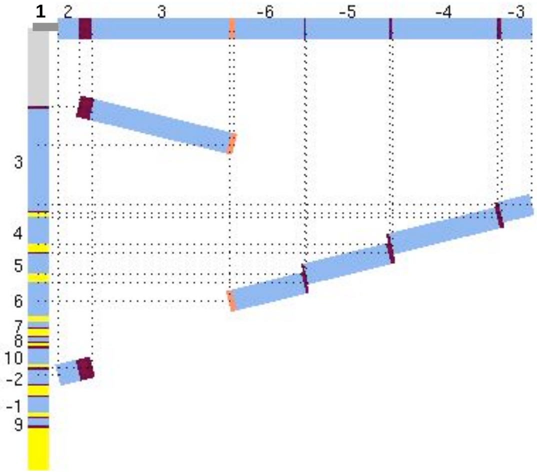

Mn2001-13

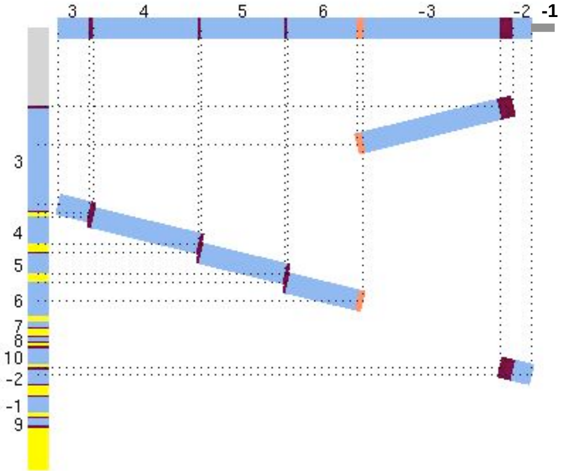

Mn2001-15

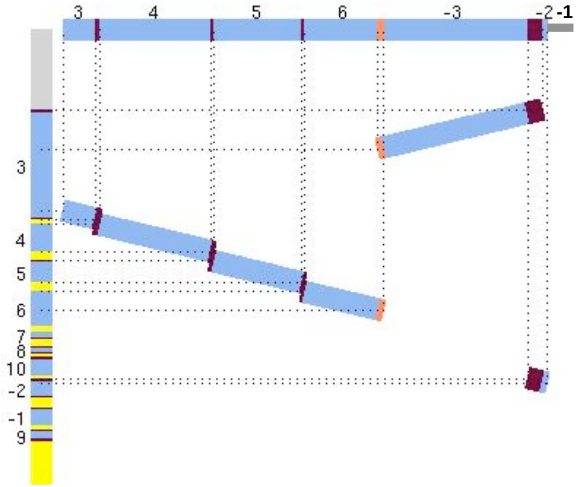

Mn2001-16

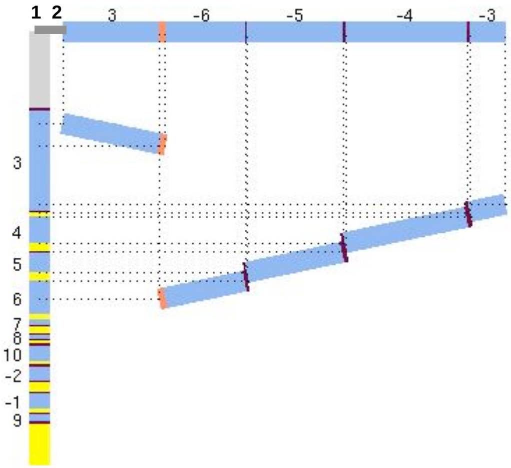

Mn2001-29

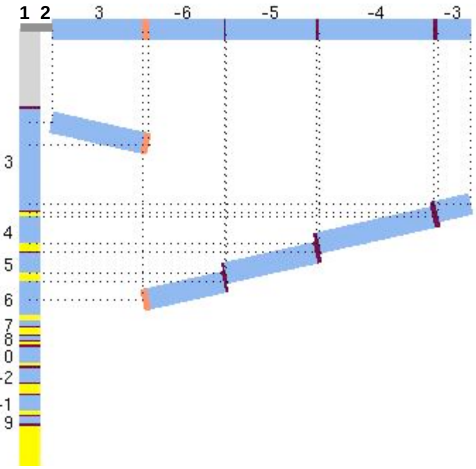

Mn2001-52

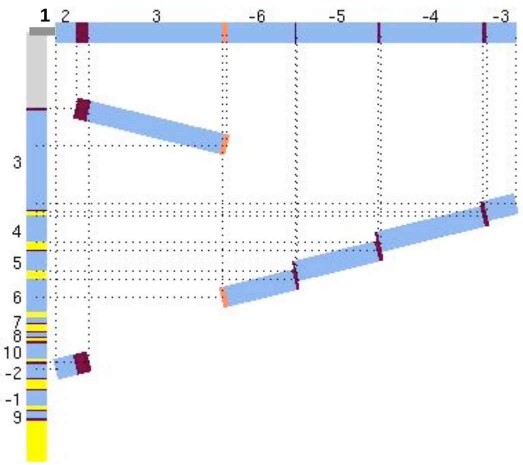

Case d:

Mn2001-5

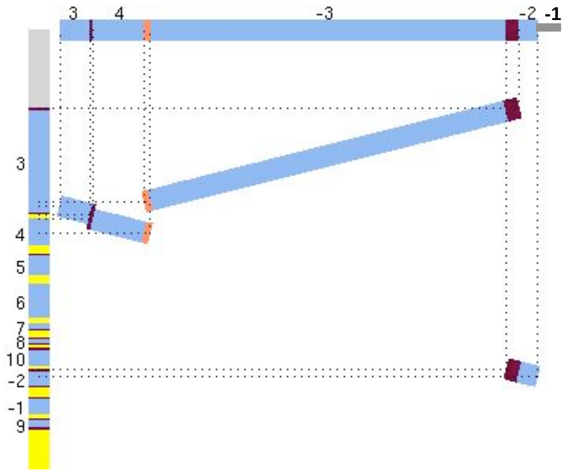

Mn2001-1

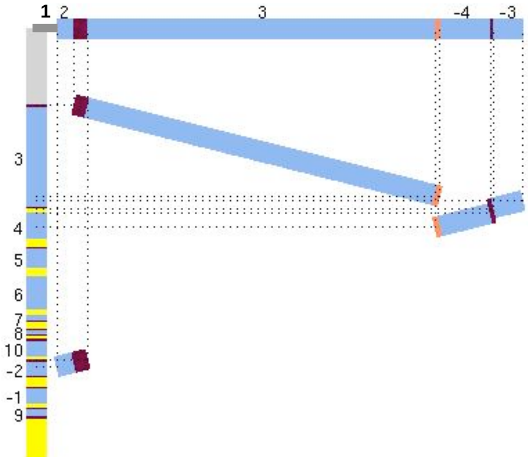

Mn2001-8

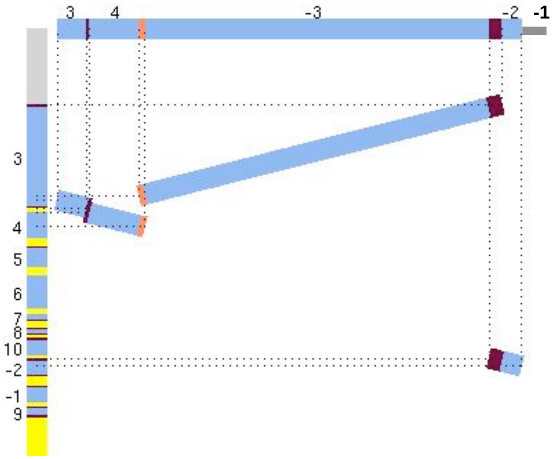

Mn2001-7

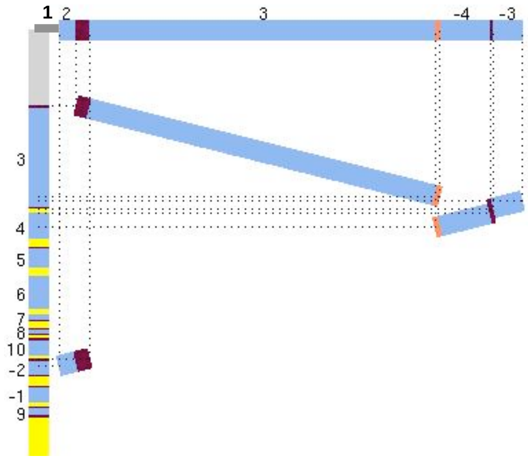

Mn2001-49

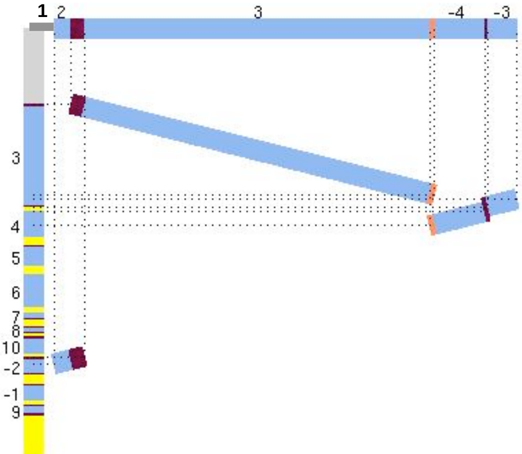

Case e:

Mn23606-4T7

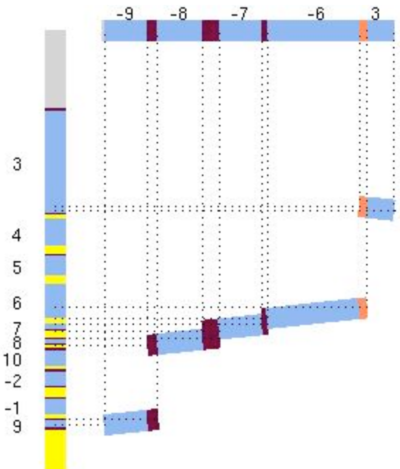

73141-T7

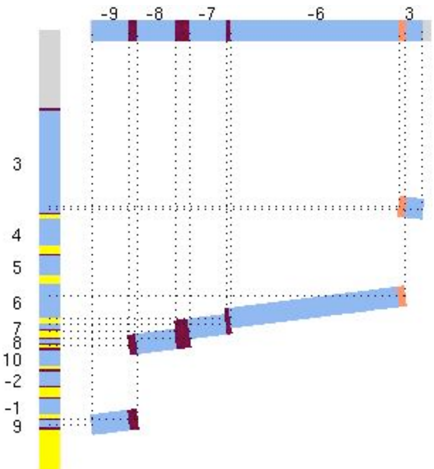

731713-T7

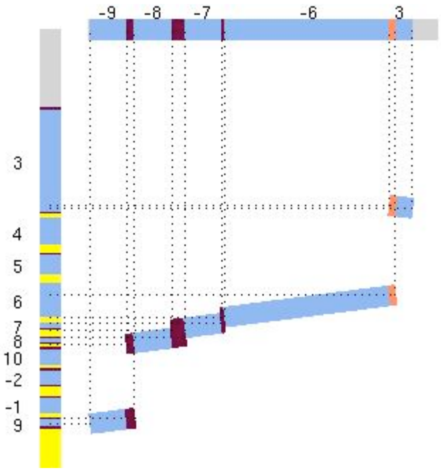

732319-T7

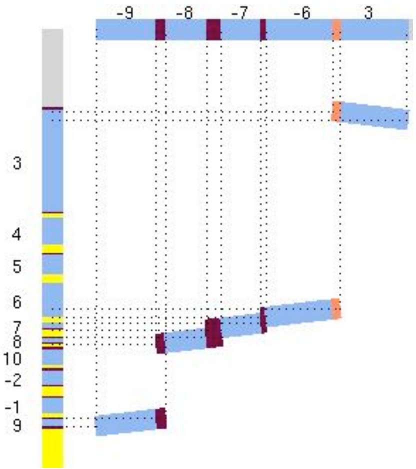

Case f:

Mn23606-16T7

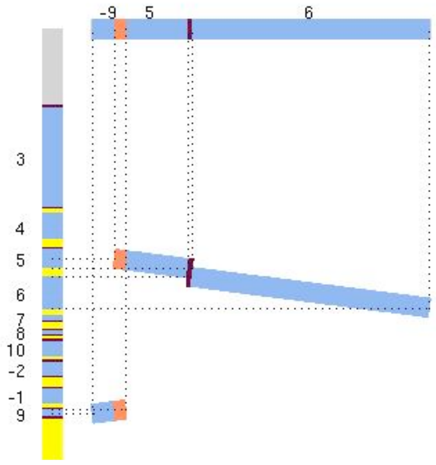

Case g:

Mn2001-59

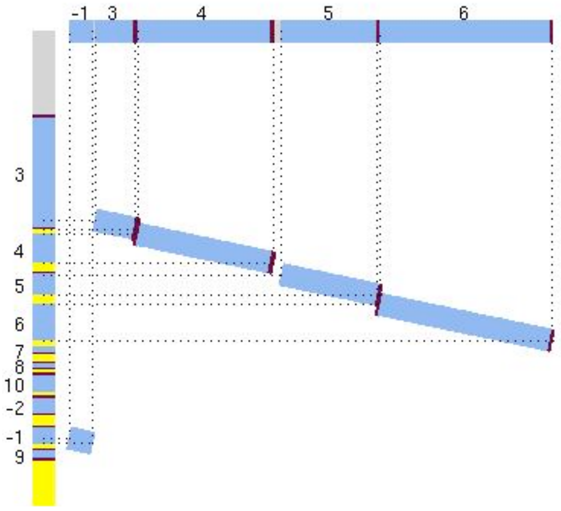

Case h:

Mn2511-1T7

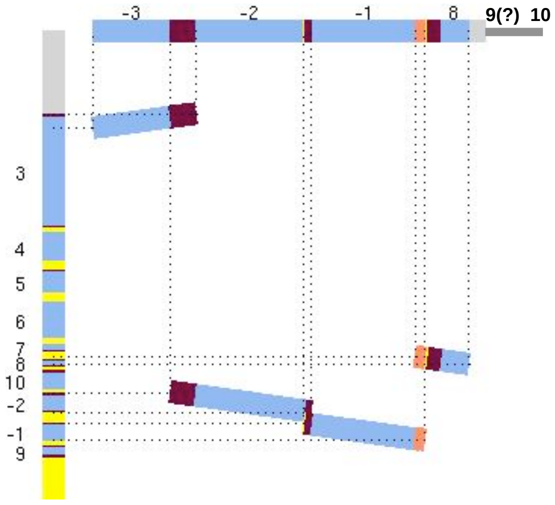

[ᄅɯ⇂ɯm9] translocation or [m3-8m10] inversion

Case i:

73155-T7

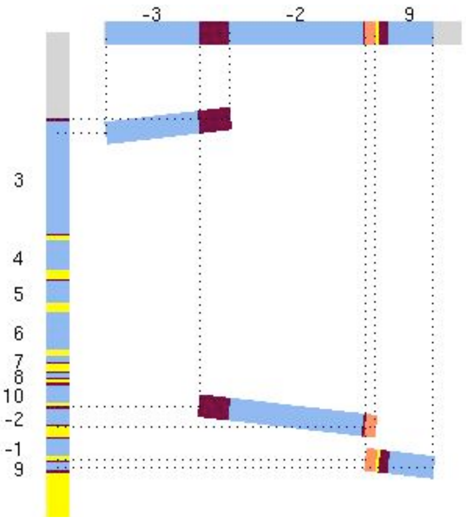

Mn19005-11-T7

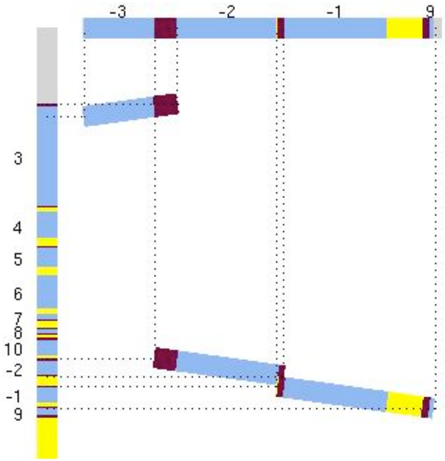

732422-T7

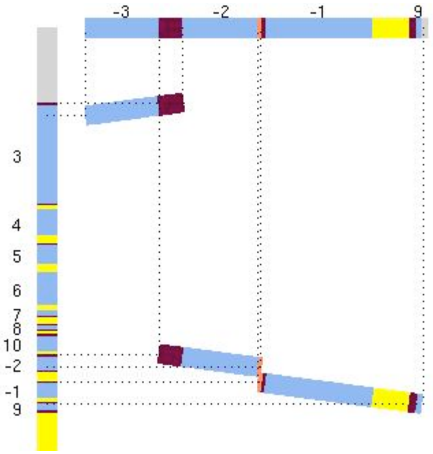

732216-T7

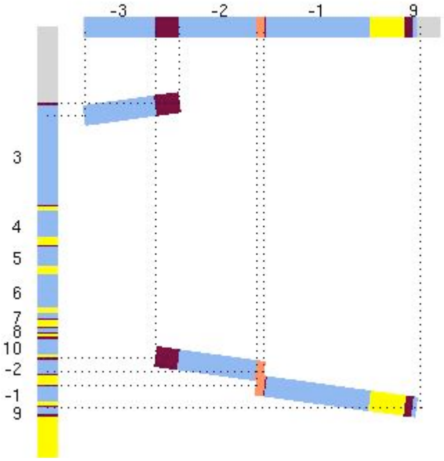

m9 translocations

Case j:

Mn180805-14

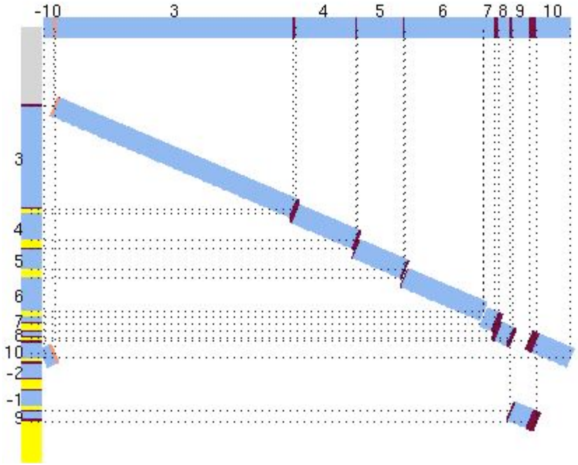

Case k:

Mn2511-17

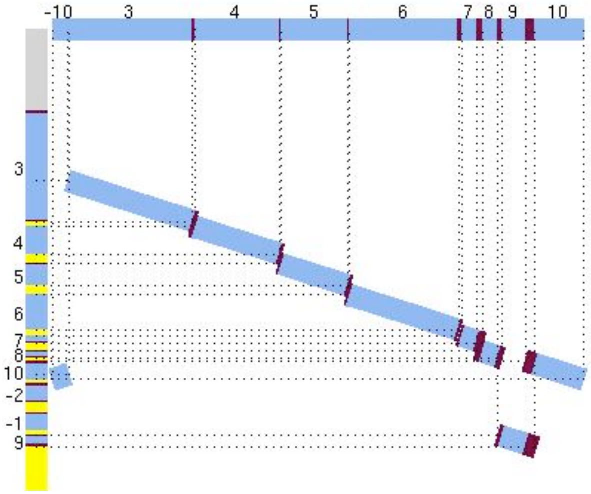

Mn7905-23

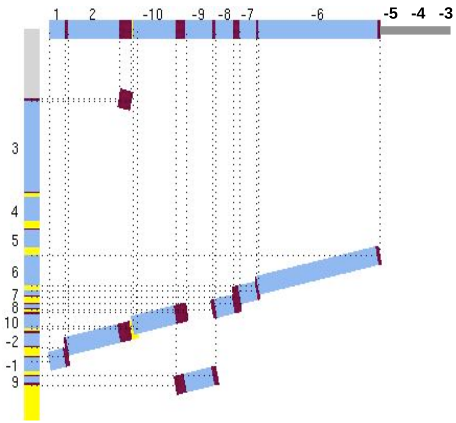

Mn101005-20

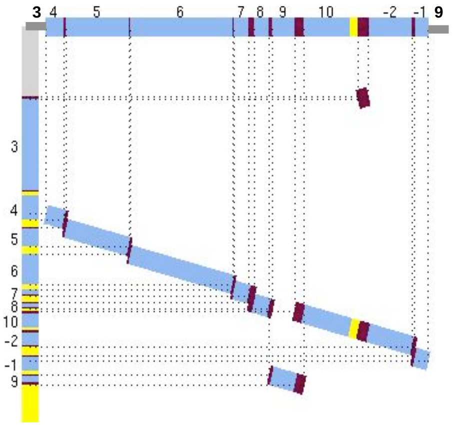

Mn101005-14

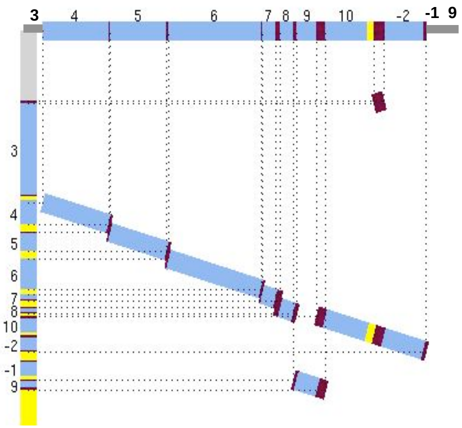

Supplement: Figure S5 — A dot-plot-like representation of all isolated aberrantly rearranged S. lemnae actin I sequences (summarized in Figure 5). The micronuclear structure is represented vertically on the Y-axis. Each molecule is represented horizontally on the X-axis. The matched regions are showed as diagonal lines. The sequences are color-coded in the same way as in Figure 1. The gray lines on the X-axis represent sequences that may be included in the first round of PCR before nested PCR. (1.78 MB PDF) [file pone.0002330.s005.pdf]
