# Supplementary material for: The Pathway to Detangle a Scrambled Gene
Source: PLoS One. 2008 Jun 4;3(6):e2330. doi: 10.1371/journal.pone.0002330 (PMC2394655; doi:10.1371/journal.pone.0002330)
Supplement: Table S1 — A summary of PCR results from different primer pairs at different developmental stages for (A) S. lemnae actin I, (B) O. trifallax actin I and (C) O. trifallax TEBPα. The ability of each primer pair to amplify micronuclear (MIC) and macronuclear (MAC) genomic sequences is indicated. The “+” and “−” signs indicate whether partially processed products can be observed among the PCR products, based on either agarose gel analysis or sequencing results. (0.09 MB DOC) [file pone.0002330.s008.doc]

A.

| **Primer Pairs*** | **Ability to amplify MIC and MAC specific products** | **Amplification of partially processed products** | | | | |
| --- | --- | --- | --- | --- | --- | --- |
|  |  | **Early** | **Early-Middle** | **Middle** | **Middle-Late** | **Late** |
| P1-P7 | Both | - | + | + | - | - |
| P5-P9 |  | + | + | + | - | - |
| P5-P31rev |  | + | + | + | + | - |
| P5-P36 |  | + | + | + | + | - |
| P5-P35 |  | + | + | + | + | - |
| F1-F4 | MIC only | + | + | + | + | - |
| F3-F6 |  | + | + | + | + | - |
| F1-P36 |  | + | + | + | + | - |
| P2rev-F6 |  | + | + | + | + | - |
| P33rev-P38rev |  | + | + | + | + | - |
| P32-P21rev | MIC only | + | + | + | + | + |
| P5-P2 |  | + | + | + | + | + |
| P5-P33 |  | + | + | + | + | + |
| P5-P21rev |  | + | + | + | + | + |
| P5-P20rev |  | + | + | + | + | + |
| P36-P5rev | Neither | - | - | - | + | + |
| P36-P7 |  | - | - | - | + | + |
| P36-P39rev |  | - | - | - | + | + |
| P36-P41rev |  | - | - | - | + | + |
| P35-P5rev |  | - | - | - | + | + |
| P35-P7 |  | - | - | - | + | + |
| P35-P39rev |  | - | - | - | + | + |
| P35-P41rev |  | - | - | - | + | + |

B.

| **Primer Pairs** | **Ability to amplify MIC and MAC specific products** | **Amplification of partially processed products** | | | | |
| --- | --- | --- | --- | --- | --- | --- |
|  |  | **0 hr** | **10 hr** | **25 hr** | **40 hr** | **55 hr** |
| MDS3F1-MDS1R1 | MIC only | - | - | + | + | - |
| MDS3F1-IES6R |  | - | - | - | - | - |
| MDS3F2-IES4R |  | - | - | + | + | - |
| MDS1R1-MDS9R | Neither | - | - | - | + | - |
| MDS8R1-MDS9R |  | - | - | - | + | - |

C.

| **Primer Pairs** | **Ability to amplify MIC and MAC specific products** | **Amplification of partially processed products** | | | | |
| --- | --- | --- | --- | --- | --- | --- |
|  |  | **0 hr** | **10 hr** | **25 hr** | **40 hr** | **55 hr** |
| MDS3R–IES7L | MIC only | - | + | + | + | - |
| IES7R-MDS17L |  | + | + | + | + | - |
| IES1R-M2L |  | - | - | + | + | - |
| MDS1R-IES11L |  | - | + | + | + | - |
| IES3R-MDS2L |  | - | - | - | - | - |
| MDS3R-IES11L |  | - | - | + | + | - |
| MDS2R-IES3L | Neither | - | + | + | + | - |
| IES3R-IES7L* |  | - | - | - | + | - |

*The final PCR results derive from nested PCR. Inner primer pairs are listed. All products in part A are nested PCR.
