# Supplementary material for: The Pathway to Detangle a Scrambled Gene
Source: PLoS One. 2008 Jun 4;3(6):e2330. doi: 10.1371/journal.pone.0002330 (PMC2394655; doi:10.1371/journal.pone.0002330)
Supplement: Table S2 — All cryptic pointers found in S. lemnae actin I molecules with unusual deletions or aberrant rearrangements. These are grouped based on whether the molecules have (A) deletion without permutation or (B) deletions with aberrant permutation (wrong MDS order). For the cryptic pointers in the first category, (A) lists the deleted IESs (all or partial), the MDS segments that are fused at the cryptic pointer, the number of times that each junction was observed, and the authentic 5′ and 3′ pointers that lie closest to the cryptic pointers in the germline sequence. For the cryptic pointers at aberrantly ordered junctions, (B) lists the observed rearrangements (joining MDS segments x to y), the number of times that each molecule type was observed, and the authentic pointers that the cryptic pointers replace. In both (A) and (B), underlined nucleotides in the cryptic pointers are contained as a sub-string of the authentic (replaced) pointer, or vice-versa. Double underline is used for overlap when more than one cryptic pointer is a substring of the same actual pointer. Boldfaced nucleotides also overlap in position, i.e. the cryptic pointer used all or part of a real pointer on at least one side of the junction. In cases indicated by *, the actual position is a telomere addition site; therefore there are no neighbouring authentic pointers. (0.08 MB DOC) [file pone.0002330.s009.doc]

A.

| **IES**  ***i*** | **MDS**  ***x-y*** | **Cryptic pointer** | **Number observed** | **Nearest 5' pointer** | **Nearest 3' pointer** |
| --- | --- | --- | --- | --- | --- |
| 1 | 3-4 | GCTTTG | 5 | **T**CGTT | **TC**G**TT** |
|  |  | **TT**GCT**T** | 4 |  |  |
|  |  | AA**TC** | 1 |  |  |
|  |  | A | 1 |  |  |
| 2 | 4-5 | AATTT | 4 | AAGA | AAGA |
|  |  | TCC | 3 |  |  |
|  |  | TCCT | 3 |  |  |
| 4 | 6-7 | **GC**AT | 10 | TGC | T**GC** |
| 7 | 10-$\overline{2}$ | ATTTAT | 4 | *telomere** | **AAA**_**CCA**GCCTTGAC(G/T)ACTC |
|  |  | **CCA** | 4 |  |  |
|  |  | TTAT | 1 |  |  |
|  |  | **AAA** | 1 |  |  |
|  |  | **AAACC** | 1 |  |  |
| 8 | 2-1 | GAGAT | 5 | ATATG | ATATG |
| 3-7 | 5-$\overline{2}$ | **CTTGAC** | 3 | ATT | AAACCAGC**CTTGAC**GACTC |
| 1-6 | 3-10 | TATTAAAT | 1 | TCGTT | GAATCA |
| 4-7 | 6-$\overline{2}$ | **CCAGCC** | 5 | TGC | AAA**CCAGCCTTGACGA**CTC |
|  |  | **CCA**(C/G)**CCTT** | 1 |  |  |
|  |  | **TGACGA** | 1 |  |  |

**B.**

| **Case** | **MDS *x-y*** | **Cryptic pointer** | **Number observed** | **Replaces pointer in MDS *x*** | **Replaces pointer in MDS *y*** |
| --- | --- | --- | --- | --- | --- |
| a, b | $\overline{8}$-3 | GGTAAGGAGC | 11 | GTTAATTTAT | GAGT(C/A)GTCAAGGCTGGTTT |
| c | 3-$\overline{6}$ | TGGAACC | 6 | TCGTT | TGC |
| d | 3-4 | CCTTACCG | 5 | TCGTT | AAGA |
| e | $\overline{3}$-6 | TCACC | 4 | GAGT(C/A)GTCAAGGCTGGTTT | ATT |
| f | $\overline{9}$-5 | GAAGAG(A/G)C | 1 | GAATCA | AAGA |
| g | $\overline{1}$-3 | none | 1 | *telomere** | GAGT(C/A)GTCAAGGCTGGTTT |
| h | $\overline{8}$-1 | TAGAGAG | 1 | GTTAATTTAT | *telomere** |
| i | $\overline{2}$-9 | **ATG**TCT | 1 | AT**ATG** | GAATCA |
| j | $\overline{10}$-3 | **TGG** | 1 | GCCAAGGACAGGTTGAA | GAGT(C/A)GTCAAGGC**TGG**TTT |
| k | $\overline{10}$-3 | A | 1 | GCCAAGGACAGGTTGAA | GAGT(C/A)GTCAAGGCTGGTTT |
