# Supplementary material for: The Pathway to Detangle a Scrambled Gene
Source: PLoS One. 2008 Jun 4;3(6):e2330. doi: 10.1371/journal.pone.0002330 (PMC2394655; doi:10.1371/journal.pone.0002330)
Supplement: Table S3 — Robustness analysis of S. lemnae actin I nonscrambled pointers (at conventional junctions). The lengths of the pointer and the IES (i) between MDS x and y are listed. Ncovered: number of junctions that are covered in the assayed sequences; Nexcised: number of junctions with an excision event; Ncorrect: number of junctions with an excision event at the correct pointer. *IES length excludes pointers. †Assuming that the accuracy of excision at different boundaries is independent, and that the error rates are not highly biased, we can roughly estimate the fraction of molecules that would be correctly-processed at all conventional IES sites by multiplying the values (Ncorrect/Nexcised) in the last row of Table S3. Based on this approximation, most (∼78%) molecules might be expected to contain at least one incorrect deletion event during development. (0.03 MB DOC) [file pone.0002330.s010.doc]

| **Pointer (*x-y*)** | ***3-4*** | ***4-5*** | ***5-6*** | ***6-7*** | ***7-8*** | ***1-2*** |
| --- | --- | --- | --- | --- | --- | --- |
| **Pointer length (bp)** | 5 | 4 | 3 | 3 | 10 | 5 |
| **IES *i*** | *1* | *2* | *3* | *4* | *5* | *8* |
| **IES length* (bp)** | 23 | 49 | 46 | 29 | 39 | 64 |
| **N_excised_/N_covered_ (%)** | 77 (47/61) | 79 (50/63) | 81 (50/62) | 78 (28/36) | 83 (29/35) | 65 (11/17) |
| **N_correct_/N_excised_ (%)** | 77 (36/47) | 80 (40/50) | 100 (50/50) | 64 (18/28) | 100 (29/29) | 55 (6/11) |
