# Supplementary material for: The Pathway to Detangle a Scrambled Gene
Source: PLoS One. 2008 Jun 4;3(6):e2330. doi: 10.1371/journal.pone.0002330 (PMC2394655; doi:10.1371/journal.pone.0002330)
Supplement: Table S4 — Robustness analysis of S. lemnae actin I scrambled pointers at permuted junctions. Lengths of scrambled pointers between MDS x and y are listed. Nprocessed: number of junctions with a rearrangement event; Ncorrect: number of junctions with a rearrangement event at the correct pointer. (0.03 MB DOC) [file pone.0002330.s011.doc]

| **Pointer (x-y)** | ***2-3*** | ***8-9*** | ***9-10*** |
| --- | --- | --- | --- |
| **Pointer length (bp)** | 19 | 6 | 17 |
| **N_correct_/N_processed_ (%)** | 48 (16/33) | 95 (20/21) | 100 (4/4) |
