# Supplementary material for: The Pathway to Detangle a Scrambled Gene
Source: PLoS One. 2008 Jun 4;3(6):e2330. doi: 10.1371/journal.pone.0002330 (PMC2394655; doi:10.1371/journal.pone.0002330)
Supplement: Method S1 — (0.05 MB DOC) [file pone.0002330.s012.doc]

# Methods S1

# PCR primers

Oligonucleotides for *S. lemnae* were purchased from MWG Biotech (Ebersberg, Germany). Oligonucleotides for *O. trifallax* were purchased from IDT Biotech (Iowa, USA). The following primer pairs were used in the PCR time-course for the three genes respectively (parentheses indicate the region of sequence where the primer is located) and their sequence details are provided below. Details of the primer positions are illustrated in Figures S1, S3, and S4.

Primer pairs used for *S. lemnae* actin I gene:

F1 (5´ flanking) – P35 (MDS 9)

F1 (5´ flanking) – P36 (MDS 9)

F1 (5´ flanking) – F4 (3´ flanking)

F3 (5´ flanking) – F6 (3´ flanking)

P32 (MDS 3) – P21rev (MDS 1)

P32 (MDS 3) – P36 (MDS 9)

P32rev (MDS 3) – P31rev (MDS 10)

P1 (MDS 3) – P7 (MDS 4)

P5 (MDS 3) – P9 (MDS 8)

P5 (MDS 3) – P2 (MDS 2)

P5 (MDS 3) – P33 (MDS 2)

P5 (MDS 3) – P21rev (MDS 1)

P5 (MDS 3) – P20rev (MDS 1)

P5rev (MDS 3) – P31rev (MDS 10)

P5rev (MDS 3) – P35 (MDS 9)

P5rev (MDS 3) – P36 (MDS 9)

P7 (MDS 4) – P35 (MDS 9)

P7 (MDS 4) – P36 (MDS 9)

P39rev (MDS 4) – P35 (MDS 9)

P39rev (MDS 4) – P36 (MDS 9)

P41rev (MDS 6) – P33rev (MDS 2)

P41rev (MDS 6) – P20 (MDS 1)

P41rev (MDS 6) – P35 (MDS 9)

P41rev (MDS 6) – P36 (MDS 9)

P31rev (MDS 10) – telomere

P2rev (MDS 2) – F6 (3´ flanking)

P33rev (MDS 2) – P38rev (3´ flanking)

1. ***MDS-specific primers***

P1 5´-CTCCAGCGCTTTGATCGGAGT-3´

P2 5´-TTGATAATGGATCCGGAGTCGTCAAG-3´

P2rev 5´-CTTGACGACTCCGGATCCATTATCAA-3´

P5 5´-GCTGAGATGGAAATCGTTAG-3´

P5rev 5´-CTAACGATTTCCATCTCAGC-3´

P7 5´-TCGTGGGATTGCTTAAGGGC-3´

# P9 5´-AGGGCTCTTTAGCTTGTCAATTC-3´

P20 5´-ACATCTAATACTCTCTATAAATC-3´

P20rev 5´-GATTTATAGAGAGTATTAGATGT-3´

P21rev 5´-AGCAAGGATATTTAAGTAAGGGC-3´

P31rev 5´-TTGATGGAATTTAGTATAAATAAGTGG-3´

P32 5´-CAATCGTTGGTAGACCCAAGAAC-3´

P32rev 5´-GTTCTTGGGTCTACCAACGATTG-3´

P33 5´- CATTATCAATAACGCAAGTTTGTTGA-3´

P33rev 5´-TCAACAAACTTGCGTTATTGATAATG-3´

P35 5´-GCCATAGTATCAGTATTGAAGAG-3´

P36 5´-GAACAWATCATCAGCCATAGTA -3´

P38rev 5´-AAGTATGYTTAARATTTGGTTTCAACC-3´

P39rev 5´-CACCGTCTGGAAGTTCGTAGT-3´

P41rev 5´-CGATTTGGACTCCGTGCTC-3´

***b) flanking primers***

F1 5´-AAAATACTTTATATTTCTCTATAGG-3´

F3 5´-ATTAAAAATTTAAATAAGCTATYTCG-3´

F4 5´-TATTATAGAGTTACAAAAATATTAG-3´

F6 5´-TTTGATTATRGCAATTTAAGTATG-3´

***c) UFW primers***

3´ end:

5´-ACTCAAGGTAAGGAGCCCTC-3´

5´-GTGCCCTATCTCGTTTATTCNNNNNNNNNN-3´

5´-GCCAAGGACAGGTTGAATGAGGT-3´

5´-CCTTGACGACTCCGGATCCATT-3´

5´-GGACAGGTTGAATGAGGTTGGTGAG-3´

5´-CGACTCCGGATCCATTATCAATAACGCAAG-3´

5´ end:

5´-GACTTTGGTGCTCTGTTTTCNNNNNNNNNN-3´

5´-TCCGTGCTCGTCATAGTCTTCCTTG-3´

5´-TCTCTTCTGACATCAACATCGCACTCTT-3´

5´-CTGTCTGGGCTGGCAATAACTTTGAC-3´

5´-TGATGTCTCTAACGATTTCCATCTCAGCTG-3´

5´-CTTTGACAGTAATGGACTTTGGTGCTCTG-3´

5´-GTGAACACGTATCCTCTTTCGGTAAG-3´

Primer pairs used for *O. trifallax* actin I gene:

MDS3F1 (MDS 3) – MDS1R1 (MDS 1)

MDS3F1 (MDS 3) – IES4R (IES 4)

MDS3F2 (MDS 3) – IES6R1 (IES 6)

MDS1R1 (MDS 1) – MDS9R (MDS 9)

MDS8R (MDS 8) – MDS9R (MDS 9)

1. ***MDS-specific primers***

MDS3F1 5´-ATTCCCATCAATCGTCGGAAGACCC-3´

MDS3F2 5´-CCCCACTCAACCCAAAGACTAACAGAG-3´

MDS1R1 5´-TGCCGCAATCGACTCTTTTAATCAGTC-3´

MDS9R 5´-GAAATACTAAGACTGTGCACACATTATAAAGATCTC-3´

MDS8R 5´-CAACCCGTCCATAGCCAATTGG-3´

1. ***IES-specific primers***

IES4R 5´-TTCCTCAAAATATTGACAACAAAAATGGTGG-3´

IES6R1 5´-GTCAATATCTCAAAATAAAATATTCTAGATTTCGTAGTTTTCC-3´

Primer pairs used for *O. trifallax* TEBPα gene:

MDS1R (MDS1) – IES11L (IES11)

MDS3R (MDS3) – IES7L (IES7)

IES1R (IES1) – MDS2L (MDS2)

IES7R (IES7) – MDS17L (MDS17)

MDS3R (MDS3) – IES11L (IES11)

MDS2R (MDS2) – IES3L (IES3)

IES7R (IES7) – IES4L (IES4)

IES7R (IES7) – IES3L (IES3)

1. ***MDS-specific primers***

MDS1R 5'-AAGATAATTGAGATTCTCGTTT-3'

MDS2R 5’-GCCCAACACTTCTACGC-3’

MDS2L 5'-CGTAATCGCTGTTATCACC-3'

MDS3R 5'-TACGCAAAGAGATTCGAAG-3'

MDS17L 5'-GGGTTATCAAAACTGTTAGAGA-3'

1. ***IES-specific primers***

IES1R 5'-TGAGATTATTACTTTGTCTTGT-3'

IES3L 5'-AAATGATAAAGTTTTCTTTTACT-3'

IES4L 5'-AAATATTTTAATAGCATTGGAA-3'

IES7L 5'-TAATGCTTTCATATTTTAACAC-3'

IES7R 5’-TAGAAGTACTAAATTTCATCC-3'

IES11L 5'-TGAGTTCTTACCTATGACTTAC-3'

**PCR reactions:**

For *S. lemnae*, all partially processed molecules were first amplified using a specific set of PCR primers and 50-100ng DNA at a specific development stage, then re-amplified either using the same primers or a pair of nested PCR primers. The development stage of the template DNA and the primers used in the PCR for each individual molecule are listed below:

**Clone name Cloned PCR primer set Initial PCR primer set Developmental stage PCR protocol**

**(nested primers) of isolation**

Mn200120T7 P5-P33 P5-P21rev early B

SacT5T7 P5-P9 P5-P9 early B

SacT4T7 P5-P9 P5-P9 early B

SacT12T7 P5-P9  P5-P9 early B

SacT34T7 P1-P7 P5-P9 early-middle B

SacT3T7 P5-P9 P5-P9 early-middle B

SacT1T7 P5-P9 P5-P9 early-middle B

SacT2T7 P5-P9 P5-P9 early-middle B

Mn2205-2T7 P32-P36 F1-P36 middle B

SacT35T7 P1-P7 P1-P7 middle B

SacT6T7 P5-P9 P5-P9 middle B

Mn200705-2T7 F1-P35 F1-P36 middle B

Ph6-SP6 F3-F6 F1-F4 middle-late A

24T7 F3-F6 F1-F4 middle-late A

63SP6 F3-F6 F1-F4 middle-late A

6SP6 F3-F6 F1-F4 middle-late A

47SP6 F3-F6 F1-F4 middle-late A

31 F3-F6 F1-F4 middle-late A

38 F3-F6 F1-F4 middle-late A

48 F3-F6 F1-F4 middle-late A

Mn101005-2 P33rev-P38rev P2rev-F6 middle-late A

Mn101005-4 P33rev-P38rev P2rev-F6 middle-late A

Mn200132T7 P5-P33 P5-P21rev middle-late B

Mn200121T7 P5-P33 P5-P21rev middle-late B

Mn10019T7 P5-P2 P5-P21rev middle-late B

Mn100111T7 P5-P2 P5-P21rev middle-late B

Mn100112T7 P5-P2 P5-P21rev middle-late B

Mn20013T7 P5-P33 P5-P21rev middle-late B

Mn200125T7 P5-P33 P5-P21rev middle-late B

Mn200126T7 P5-P33 P5-P21rev middle-late B

Mn200146T7 P5-P33 P5-P21rev middle-late B

Mn20016T7 P5-P33 P5-P21rev middle-late B

Mn10017T7 P5-P2 P5-P21rev middle-late B

Mn20012T7 P5-P33 P5-P21rev middle-late B

Mn200134T7 P5-P33 P5-P21rev middle-late B

Mn200161T7 P5-P33 P5-P21rev middle-late B

Mn200156T7 P5-P33 P5-P21rev middle-late B

Mn200110T7 P5-P33 P5-P21rev middle-late B

Mn200135T7 P5-P33 P5-P21rev middle-late B

Mn200136T7 P5-P33 P5-P21rev middle-late B

Mn200131T7 P5-P33 P5-P21rev middle-late B

Mn200133T7 P5-P33 P5-P21rev middle-late B

Mn200127T7 P5-P33 P5-P21rev middle-late B

Mn100110T7 P5-P2 P5-P21rev middle-late B

Mn200128T7 P5-P33 P5-P21rev middle-late B

Mn200130T7 P5-P33 P5-P21rev middle-late B

Mn200112T7 P5-P33 P5-P21rev middle-late B

Mn200111T7 P5-P33 P5-P21rev middle-late B

Mn200122T7 P5-P33 P5-P21rev middle-late B

Mn20019T7 P5-P33 P5-P21rev middle-late B

Mn200158T7 P5-P33 P5-P21rev middle-late B

Mn200119T7 P5-P33 P5-P21rev middle-late B

Mn100116T7 P5-P2 P5-P21rev middle-late B

Mn7905-10 P5-P33 P32-P21rev middle-late B

Mn101005-13 P5-P21rev F1-P36 middle-late A

Mn101005-16 P5-P21rev F1-P36 middle-late A

Mn101005-19 P5-P21rev F1-P36 middle-late A

Mn101005-21 P5-P21rev F1-P36 middle-late A

Mn101005-22 P5-P21rev F1-P36 middle-late A

Mn101005-24 P5-P21rev F1-P36 middle-late A

Mn101005-9 P5-P21rev F1-P36 middle-late A

Mn200117T7 P5-P33 P5-P21rev late B

Mn200118T7 P5-P33 P5-P21rev late B

Mn200154T7 P5-P33 P5-P21rev late B

Mn200124T7 P5-P33 P5-P21rev late B

Mn10018T7 P5-P2 P5-P21rev late B

Mn200147T7 P5-P33 P5-P21rev late B

Mn200148T7 P5-P33 P5-P21rev late B

Mn100113T7 P5-P2 P5-P21rev late B

Mn100114T7 P5-P2 P5-P21rev late B

Mn100115T7 P5-P2 P5-P21rev late B

Mn100117T7 P5-P2 P5-P21rev late B

Mn100118T7 P5-P2 P5-P21rev late B

Mn200150T7 P5-P33 P5-P21rev late B

Mn200151T7 P5-P33 P5-P21rev late B

Mn101005-20 P5-P21rev F1-P36 middle-late A

Mn101005-14 P5-P21rev F1-P36 middle-late A

Mn7905-23 P5-P21rev P5-P20rev late B

Mn2001-52 P5-P33 P5-P20rev late B

Mn2001-49 P5-P33 P5-P20rev late B

Mn180805-14 P31rev-telomere anchor P31rev-telomere late B

Mn2001-59 P41rev-P20 P41rev-P33rev late B

Mn2511-17 P5-P31rev P5-P31rev middle-late B

Mn2511-1T7 P32rev-P31rev P32rev-P31rev middle-late B

Mn23606-4 P5rev-P35 P36-P5rev middle-late B

Mn23606-6 P7-P35 P7-P36 middle-late B

Mn23606-7 P7-P35 P7-P36 middle-late B

Mn23606-8 P7-P35 P7-P36 middle-late B

Mn23606-10 P7-P35 P7-P36 middle-late B

Mn23606-11 P39rev-P35 P39rev-P36 middle-late B

Mn23606-12 P39rev-P35 P39rev-P36 middle-late B

Mn23606-14 P39rev-P35 P39rev-P36 middle-late B

Mn23606-15 P39rev-P35 P39rev-P36 middle-late B

Mn23606-16 P41rev-P35 P41rev-P36 middle-late B

Mn23606-21 P5rev-P35 P39rev-P36 middle-late B

Mn23606-23 P5rev-P35 P39rev-P36 middle-late B

Mn23606-24 P5rev-P35 P39rev-P36 middle-late B

Mn2001-5 P5-P33 P5-P20rev late B

Mn2001-1 P5-P33 P5-P20rev late B

Mn2001-7 P5-P33 P5-P20rev late B

Mn2001-8 P5-P33 P5-P20rev late B

Mn2001-13 P5-P33 P5-P20rev late B

Mn2001-14 P5-P33 P5-P20rev late B

Mn2001-15 P5-P33 P5-P20rev late B

Mn2001-16 P5-P33 P5-P20rev late B

Mn2001-23 P5-P33 P5-P20rev late B

Mn2001-49 P5-P33 P5-P20rev late B

Mn19005-11-T7 P5rev-P35 P5rev-P36 middle-late B

732422-T7 P5rev-P35 P5rev-P36 middle-late B

73141-T7 P5rev-P35 P5rev-P36 middle-late B

73155-T7 P5rev-P35 P5rev-P36 middle-late B

73157-T7 P5rev-P35 P5rev-P36 middle-late B

731713-T7 P5rev-P35 P5rev-P36 middle-late B

732216-T7 P5rev-P35 P5rev-P36 middle-late B

732319-T7 P5rev-P35 P5rev-P36 middle-late B

*S. lemnae* PCR protocols:

**A. Phusion (Finnzymes Phusion High Fidelity PCR Kit)**

**First round of PCR:**

Basic 20ul reaction conditions according to manual, with 50-100ng DNA template.

98°C 30 seconds;

98°C 10 seconds, 55°C 30 seconds, 72°C 1.5 minutes, 35 cycles;

72°C 10 minutes; 4°C hold.

**Second round of PCR:**

Re-amplification with *Taq* DNA polymerase (Genecraft) (for TA cloning) using 1ul of 1:10 dilution of the first round PCR product as template.

95°C 30 seconds, 1 minute of varying temperature (according to primer annealing temperature), 74°C 1.5 minutes, 20 cycles.

**B. BioTherm *Taq* DNA polymerase (Genecraft)**

**First round of PCR:**

Basic 20ul reaction conditions according to manual, with 50-100ng DNA template.

95°C 4 minutes,

95°C 1.5 minutes, 52°C 1.5 minutes, 74°C, 1.5 minutes, 5 cycles;

95°C 30 seconds, 1 minute of varying temperature (according to primer annealing temperature), 74°C 1 minute, 30 cycles;

74°C 10 minutes.

**Second round of PCR:**

Re-amplification with the same enzyme using 1ul of 1:10 dilution of the first round PCR product as template.

95°C 30 seconds, 1 minute of varying temperature (according to primer annealing temperature), 74°C 1.5 minutes, 20 cycles.

The temperature for the annealing step was adjusted between 53 and 57°C based on the GC content of the primers.

*O. trifallax* PCR protocols:

For *O. trifallax actin I* and *TEBPα*, 200-300ng template DNA from a specific developmental time point were amplified in 50 µl 1X PCR buffer (Roche) and 10 µM each primer, using 1U *Taq* DNA polymerase (Roche) and manual hot-start via addition of polymerase and buffer at 94°C.

Standard PCR conditions for *actin I* were 30 cycles of 30 seconds 94°C, 30 seconds 55°C, and 2 minutes 68°C.

Standard PCR conditions for *TEBPα* were 25 cycles of 42 seconds 95°C, 30 seconds 48°C, and 45 seconds 65°C.

For the IES7R-IES4L primer pair, 1 µl of the PCR was diluted 1/100 in water and then amplified in a semi-nested PCR as above, with primers IES7R-IES3L.

PCR products were cloned directly into the pCR2.1 TOPO vector (Invitrogen).
