# Supplementary material for: The Pathway to Detangle a Scrambled Gene
Source: PLoS One. 2008 Jun 4;3(6):e2330. doi: 10.1371/journal.pone.0002330 (PMC2394655; doi:10.1371/journal.pone.0002330)
Supplement: Data S1 — (0.02 MB ZIP) [file pone.0002330.s013.zip › Data S1.doc]

**Supporting Data Files**:

**SlActinIConventional.fas**: Multiple sequence alignment file containing all molecules with any DNA deletions but no reordering in *S. lemnae actin I* (schematic representations in Figure 3).

**SlActinIPermuted.fas**: Sequence file containing all molecules with partial rearrangements in *S. lemnae actin I* (summary in Figure 5 and schematic representations in Figure S5).

**OtActinIConventional.fas**: Multiple sequence alignment file containing all molecules with any DNA deletions but no reordering in *O. trifallax actin I* (schematic representations in Figure S3A).

**OtActinIPermuted.fas**: Sequence file containing all molecules with partial rearrangements in *O. trifallax actin I* (schematic representations in Figure S4A).

**OtTEBPaConventional.fas**: Multiple sequence alignment file containing all molecules with any DNA deletions but no reordering in *O. trifallax TEBPα* (schematic representations in Figure S3B);

**OtTEBPaPermuted.fas**: Sequence file containing all molecules with partial rearrangements in *O. trifallax TEBPα* (schematic representations in Figure S4B).
